# Nonadhesive functions of E-cadherin promote cancer cell survival and metastasis

**DOI:** 10.1083/jcb.202507222

**Published:** 2026-10-22

**Authors:** Ryan L. Huizar, James V. McCann, Malak Amer, Beza Woldemeskel, Daisuke Murata, Nanami Senoo, Abhijit Deb Roy, Benjamin C. Orsburn, Steven M. Claypool, Hiromi Sesaki, Miho Iijima, Namandjé N. Bumpus, Takanari Inoue, Andrew J. Ewald

**Affiliations:** 1 Department of Cell Biology, The Johns Hopkins University School of Medicine, Baltimore, MD, USA; 2 Department of Oncology, Sidney Kimmel Comprehensive Cancer Center, The Johns Hopkins University School of Medicine, Baltimore, MD, USA; 3 Giovanis Institute for Translational Cell Biology, The Johns Hopkins University School of Medicine, Baltimore, MD, USA; 4 Department of Physiology, Pharmacology and Therapeutics, Mitochondrial Phospholipid Research Center, Johns Hopkins University School of Medicine, Baltimore, MD, USA; 5 Center for Cell Analysis and Modeling and Department of Cell Biology, University of Connecticut School of Medicine, Farmington, CT, USA; 6 Organ Pathobiology and Therapeutics Institute, The University of Pittsburgh, Pittsburgh, PA, USA; 7 Department of Pharmacology and Molecular Sciences, The Johns Hopkins University School of Medicine, Baltimore, MD, USA

## Abstract

E-cadherin suppresses invasion and promotes metastasis in invasive ductal breast cancer, through suppression of reactive oxygen species (ROS). We sought to determine the cellular source of apoptosis-inducing ROS and to identify the domains of E-cadherin that are essential for cancer cell survival. Inhibition of mitochondria-derived ROS was sufficient to rescue cell death following E-cadherin deletion, in both 3D colony formation and in vivo metastasis assays. We next generated Cdh1 constructs by mutating amino acids or deleting domains, then tested their ability to rescue colony formation in E-cadherin–deleted cells. Surprisingly, E-cadherin constructs in which the extracellular domains were deleted, adhesion was compromised, or catenin interactions were blocked could rescue elevated mitochondrial ROS and 3D colony formation. Conversely, a construct lacking the cytoplasmic domain was unable to rescue ROS or cell death. Our findings reveal that E-cadherin’s ability to regulate mitochondrial ROS, cancer cell survival, and metastatic outgrowth is separable from its role in intercellular adhesion.

## Teaser

Huizar et al. show that E-cadherin promotes breast cancer cell survival and metastasis by suppressing mitochondrial ROS. The cytoplasmic domain of E-cadherin is essential, while extracellular domains, and adhesion and catenin interactions are not. Thus, E-cadherin’s regulation of mitochondrial ROS and cell survival during metastasis is independent of cell–cell adhesion.

## Introduction

Most deaths from cancer arise from metastasis, the process by which cancer cells disseminate from the primary tumor, migrate through local stroma, intravasate, travel through the bloodstream, extravasate, then colonize distant organs (Gerstberger et al., 2023; Hapach et al., 2019; Pantel and Brakenhoff, 2004). Cancer cells encounter very different microenvironments during metastasis, experience multiple types of molecular stress, and frequently die in transit (Fidler, 1970; Gerstberger et al., 2023; Massagué and Obenauf, 2016). Consequently, metastasis is an inefficient process, with a very small fraction of disseminating cancer cells successfully seeding clinically significant metastases (Cameron et al., 2000; Luzzi et al., 1998; Massagué and Obenauf, 2016). We are motivated to understand the molecular pathways that promote cancer cell survival during metastasis as a basis to develop new therapeutic strategies.

E-cadherin (CDH1) is a transmembrane protein that mediates calcium-dependent adhesion between epithelial cells. Homophilic binding of E-cadherin on adjacent epithelial cells provides the framework for normal epithelial architecture in many organs. This adhesive function is mediated through intracellular interactions with catenins, which in turn bind to and stabilize F-actin at the cell cortex (Mège and Ishiyama, 2017; van Roy and Berx, 2008). E-cadherin exhibits context-dependent dynamics at the plasma membrane due to membrane diffusion and several pathways of internalization and trafficking (Bryant and Stow, 2004; Erami et al., 2016; Rubtsova et al., 2022).

Early experiments identified genetic mutations in E-cadherin as causal in several cancer types including diffuse gastric and lobular breast cancer (Berx et al., 1995; De Leeuw et al., 1997; Derksen et al., 2006; Guilford et al., 1998; Perl et al., 1998). Epigenetic downregulation of E-cadherin is often associated with increased rates of invasion and metastasis, generally carrying a poor prognosis (Christofori and Semb, 1999; Li et al., 2017). Furthermore, modulation of E-cadherin results in reorganization of the cytoskeleton, changes to intracellular signaling pathways, and alterations to cell adhesion and motility, across several models of breast cancer (Onder et al., 2008; Schipper et al., 2019).

However, ∼90% of invasive breast cancer cases are classified as invasive ductal carcinoma (IDC) (Badowska-Kozakiewicz et al., 2017). E-cadherin expression is typically maintained in IDC in both the primary tumor and metastases (Chao et al., 2010; Chao et al., 2012), though reduced E-cadherin expression can be observed in poorly differentiated IDC samples (Li et al., 2017; Moll et al., 1993). Loss or downregulation of E-cadherin is associated with increased dissemination, but these cancer cells tend to migrate individually and exhibit extremely inefficient metastatic colonization (Massagué and Obenauf, 2016). Consistent with this observation, breast cancer cells metastasize more efficiently as clusters, as opposed to single cells (Aceto et al., 2014; Cheung and Ewald, 2016; Cheung et al., 2016). Furthermore, E-cadherin levels are often maintained or even enriched in IDC metastases when compared to their paired primary tumors (Chao et al., 2010; Chao et al., 2012; Kowalski et al., 2003).

MMTV-PyMT is a genetically engineered mouse model of breast cancer that is frequently used in metastasis studies because tumors develop rapidly, gene expression is similar to human luminal B breast cancer, and metastasis to the lungs is highly efficient (Guy et al., 1992; Herschkowitz et al., 2007). When cancer cells are isolated from this model and grown in 3D culture, cells invade collectively through activation of a basal epithelial program highlighted by K14 and p63 expression (Cheung et al., 2013). We previously showed that Cdh1 is an invasion suppressor, survival factor, and metastasis promoter in multiple murine and human models of IDC (Padmanaban et al., 2019). When E-cadherin is genetically ablated in the MMTV-PyMT model, invasion is increased but systemic dissemination and lung colonization are decreased (Padmanaban et al., 2019). E-cadherin–deleted cells exhibit increased TGF-β signaling, accumulate reactive oxygen species (ROS), and undergo apoptosis (Padmanaban et al., 2019). Accordingly, deletion of E-cadherin led to more invasion and less metastasis (Padmanaban et al., 2019). In this study, we sought to determine the cellular source of apoptosis-inducing ROS following Cdh1 deletion and to identify the domains of E-cadherin that are essential for limiting ROS and promoting cancer cell survival.

## Results

**Figure S1.**
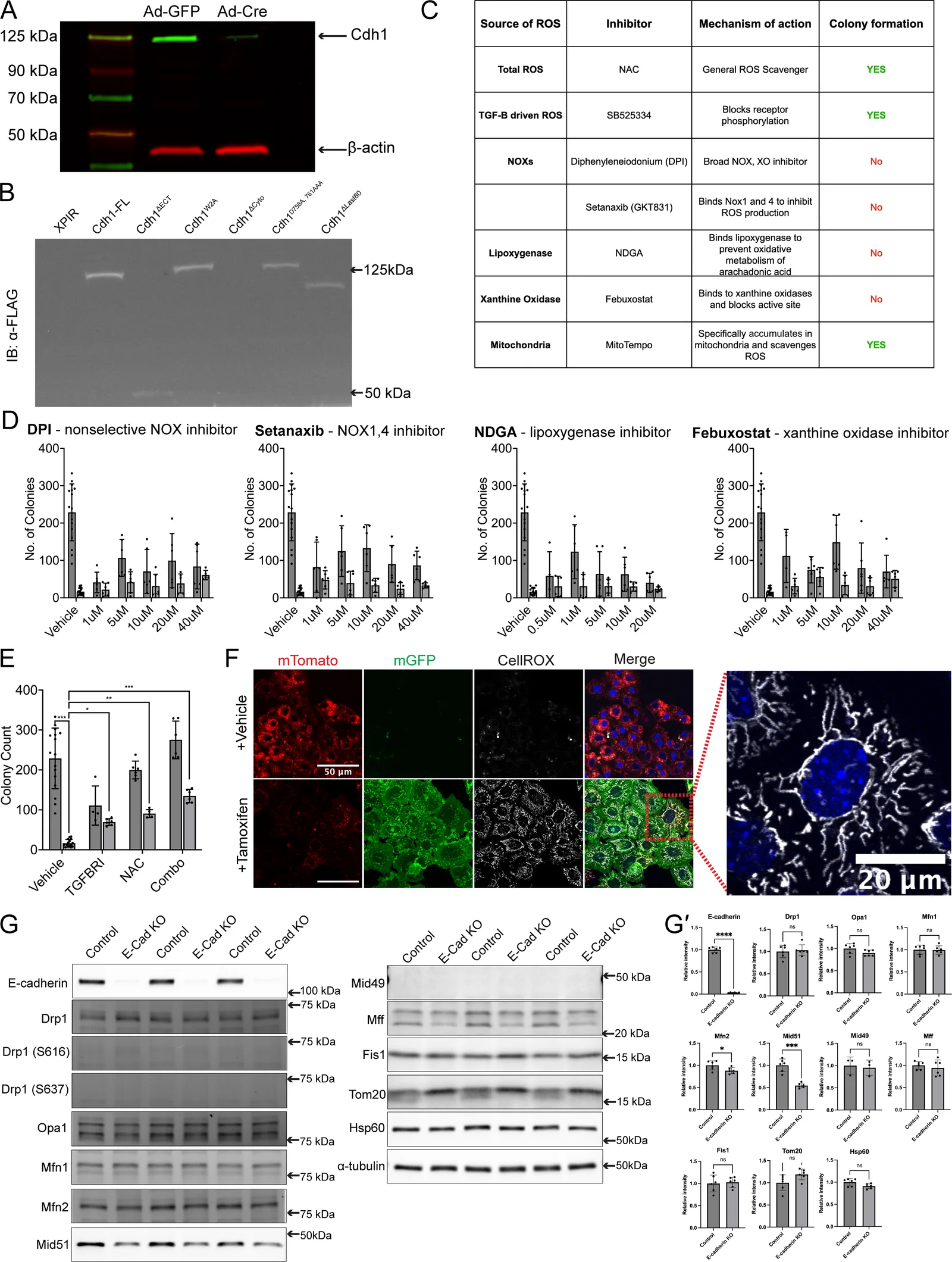
**Validation of E-cadherin manipulation and characterization of ROS sources and mitochondrial proteins. (A) **Western blot of lysates derived from MMTV-PyMT;Cdh1^fl/fl^ organoids treated with Ad-Cre or Ad-GFP. Lysates were immunoblotted for E-cadherin (ECCD-2 clone) and β-actin. **(B)** Expression of FLAG-tagged E-cadherin rescue constructs. Representative western blot of organoid-derived cells expressing the indicated FLAG-tagged E-cadherin constructs following infection with XPIR (empty vector), full-length E-cadherin, ΔEct, W2A, ΔCyto, D758A–761AA, or ΔLast80 constructs. Lysates were probed with anti-FLAG antibody to assess the relative expression of rescue constructs. **(C)** Summary of small molecules used in ROS inhibitor screen. **(D)** Dose–response for organoids treated with each ROS inhibitor, with and without intact E-cadherin. For each ROS inhibitor, *n* = 31 vehicle wells were analyzed, *n* = 12 for 1, 5, 10, 20, and 40 µM conditions. r = 2 biological replicates. Error bars represent the standard deviation. **(E)** Quantification of the colony formation assay for SB525334 (TGFBi) and NAC, positive controls confirmed to rescue in previous studies. For Ad-Cre-null–treated clusters, vehicle *n* = 16 total wells were analyzed, TGFBi *n* = 6, NAC *n* = 6, combination treatment *n* = 6. For Ad-Cre–treated clusters, vehicle *n* = 15 total wells were analyzed, TGFBi *n* = 6, NAC *n* = 6, combination treatment *n* = 6. r = 3 biological replicates. Interaction, *P < 0.05; **P <0 .01; ***P < 0.0001; Treatment (Ad-Cre vs Ad-Cre-null), ***P < 0.0001 by 2-way ANOVA with multiple comparisons. Error bars represent the standard deviation. **(F)** Scanning confocal micrograph of CellROX intensity in E-cadherin intact (top) and deleted (bottom) at high magnification (60×). **(G and G′)** Western blot analysis of mitochondrial fission, fusion, and mitochondrial mass–associated proteins in control and E-cadherin knockout cells. Lysates from control and E-cadherin knockout conditions were probed for E-cadherin to confirm efficient deletion, as well as Drp1, phospho-Drp1 (S616, S637), Opa1, Mfn1, Mfn2, Mid51/Mief1, Mid49, Mff, Fis1, Tom20, and HSP60. Relative protein abundance is quantified in the right panel. Protein intensities were normalized to α-tubulin and plotted relative to control cells. Two biological replicates were analyzed, each with technical triplicates. Statistical significance was determined by Welch’s *t* test; ns, not significant; *P < 0.05; ***P < 0.001; ****P < 0.0001. Error bars represent the standard deviation. Source data are available for this figure: SourceData FS1.

**Figure 1.**
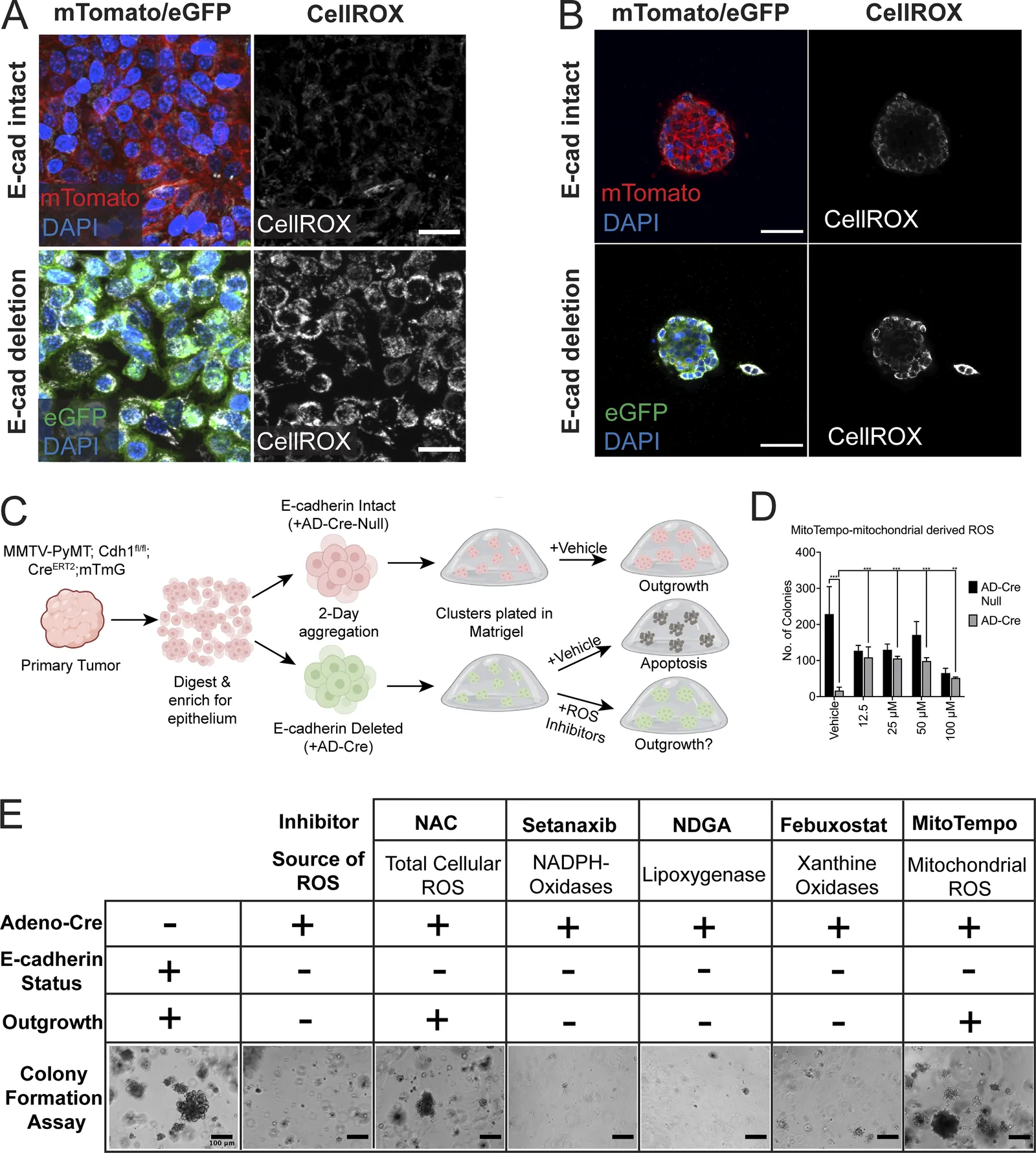
**Targeted drug screen localizes cellular source of ROS upon Cdh1 loss. (A and B)** Monolayers or (B) 3D Matrigel cultures of cancer cells derived from MMTV-PyMT;Cdh1^fl/fl^; Rosa26-mTmG mice treated with either Ad-Cre-null (E-cadherin intact) or Ad-Cre (E-cad deleted). Global ROS was detected using CellROX Deep Red reagent and imaged by scanning confocal microscopy. Nuclei were stained with DAPI. Scale bars, 50 µm. **(C)** Schematic of the experimental design for ROS inhibitor screen. Created using https://BioRender.com/or7ca5p (images isolated for use in Illustrator during figure preparation). **(D)** Quantification of the colony formation assay for MitoTempo (*n* = 55 wells across conditions, r = 3 independent donor tumors). Interaction, **P < 0.01; ***P < 0.001; Treatment (Ad-Cre vs Ad-Cre-null), ***P < 0.0001 by 2-way ANOVA with multiple comparisons. Error bars represent the standard deviation. **(E)** Overview of results of colony formation assay used in ROS inhibitor screen of MMTV-PyMT;Cdh1^fl/fl^ organoids. These images were collected from colony formation assays used to generate the broader quantification across inhibitors that is reported in Fig. S1, D and E. Colony outgrowth was imaged by DIC 5 days after seeding.

We first sought to optimize a simple assay to interrogate the mechanisms by which Cdh1 deletion leads to lethal increases in ROS. To accomplish this goal, 2D cultures of cancer cells derived from MMTV-PyMT;Cdh1^fl/fl^;Rosa26-mTmG;Cre^ERT2^ mammary tumors were treated with tamoxifen or Adeno-Cre for 48 h to induce Cre-mediated deletion of Cdh1. Deletion was confirmed by a significant reduction in E-cadherin protein levels on western blots (Fig. S1 A). The red-to-green color switch associated with the ROSA^mT/mG^ allele confirmed robust recombination upon tamoxifen or Ad-Cre treatment. To assess the impact of E-cadherin loss on ROS levels, monolayers were incubated with CellROX, a cell-permeable fluorogenic probe that fluoresces upon oxidation. Treatment with CellROX confirmed that Cdh1 deletion increased cellular ROS levels, consistent with previous findings from our group (Fig. 1 A; Padmanaban et al., 2019). Notably, loss of E-cadherin led to strongly enriched CellROX fluorescence in 2D culture (Fig. S1 F) and in 3D culture (Fig. 1 B).

Given the known connections between E-cadherin, F-actin organization (Mège and Ishiyama, 2017), and mitochondrial fission/fusion (Murata et al., 2020), we performed additional immunoblotting to determine whether E-cadherin loss alters regulators of mitochondrial dynamics. Lysates from control and E-cadherin knockout cells were blotted for E-cadherin, Drp1, phospho-Drp1 S616, phospho-Drp1 S637, Opa1, Mfn1, Mfn2, Mid51/Mief1, Mid49/Mief2, Mff, Fis1, Tom20, Hsp60, α-tubulin, and Gapdh. E-cadherin deletion led to significant effects only on the mitochondrial dynamics proteins Mid51/Mief1 and Mfn2 (Fig. S1, G and G′). Based on these data, E-cadherin loss does not broadly alter the abundance of the mitochondrial fission/fusion machinery or mitochondrial mass markers assessed here, though altered mitochondrial dynamics may still underlie aspects of the cell survival phenotype.

ROS can be produced in multiple cellular compartments by different enzymes and so we next sought to rescue cell death following Cdh1 deletion by inhibiting different classes of ROS-generating enzymes. Employing our established primary organotypic culture system (Padmanaban et al., 2020), we treated organoids derived from MMTV-PyMT;Cdh1^fl/fl^;Rosa26-mTmG;Cre^ERT2^ mammary tumors with Ad-GFP (vehicle) or Ad-Cre (to induce Cdh1 recombination). Following recombination, primary organoids were digested to clusters, embedded in Matrigel, cultured in the presence of various ROS inhibitors, then analyzed for the extent of colony formation (Fig. 1, C–E and Fig. S1, C–E). We treated with a broad antioxidant/ROS scavenger (N-acetylcysteine [NAC]), a nonselective NADPH oxidase (NOX) inhibitor (diphenyleneiodonium [DPI]), a selective NOX inhibitor (NOX1/4; setanaxib), lipoxygenase inhibitor (NDGA), xanthine oxidase inhibitor (febuxostat), and a mitochondrial ROS scavenger (MitoTempo). Only NAC and MitoTempo significantly rescued colony formation of Cdh1-deleted clusters, relative to Cdh1-intact control clusters (Fig. 1, D and E); inhibitors of NOXs, lipoxygenases, or xanthine oxidases did not rescue colony formation (Fig. S1, D and E).

**Figure 2.**
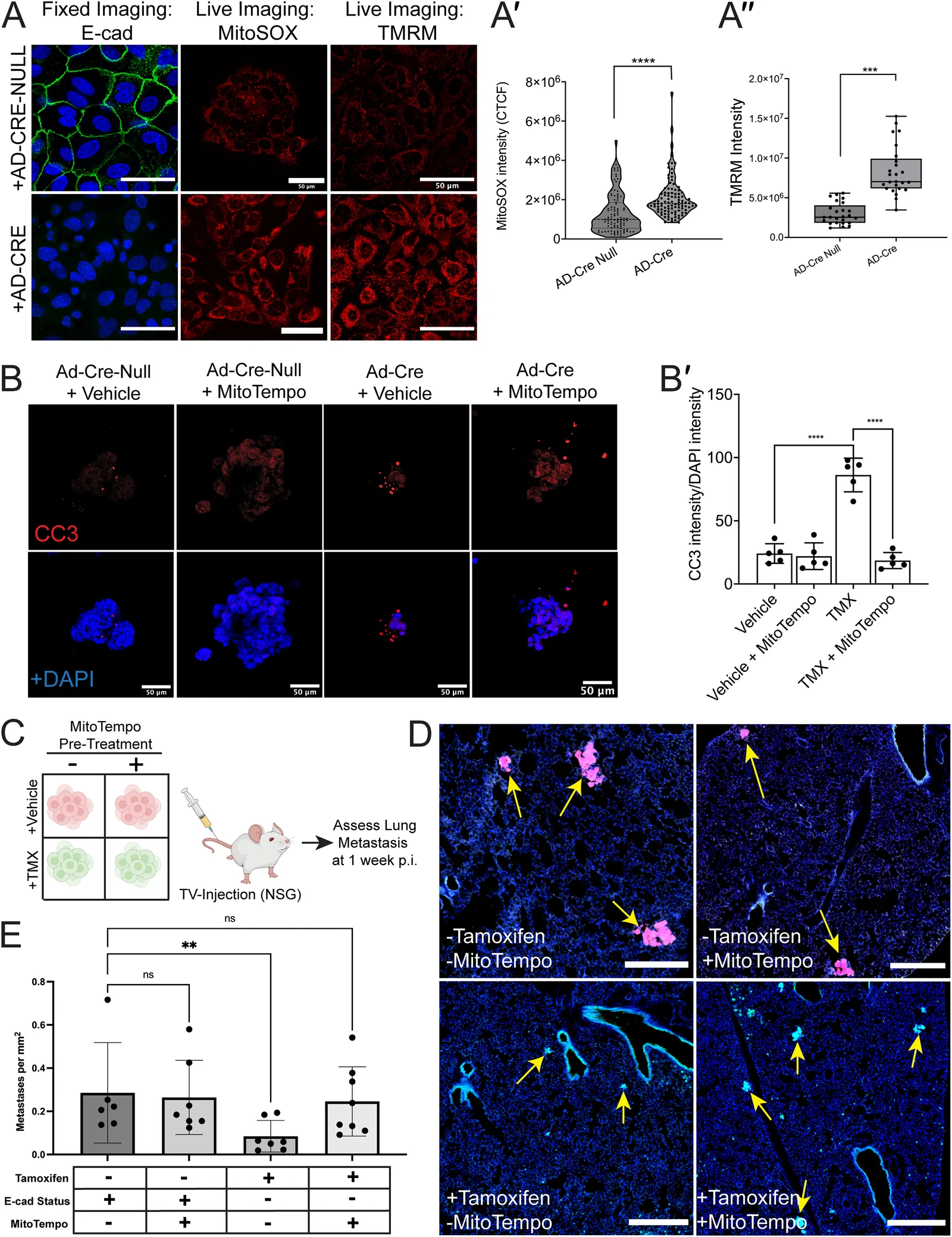
**Inhibition of mitochondrial ROS increases outgrowth capacity of Cdh1-null cells in vitro and in vivo. (A–A″)** Monolayers of cancer cells derived from MMTV-PyMT;Cdh1^fl/fl^ mice treated with Ad-Cre or Ad-Cre-null, stained with MitoSOX Red reagent or TMRM, and imaged live by scanning confocal microscopy to assess MitoSOX abundance or mitochondrial membrane potential, respectively. Subsequently, fixed monolayers were stained for E-cadherin and DAPI to confirm E-cadherin deletion by Ad-CRE. Scale bars, 50 µm. CTCF is reported for A′ MitoSOX intensity (*n* = 100 confocal micrographs of MitoSOX-stained monolayers, 50 per condition; r = 3 biological replicates) or A″ TMRM intensity (*n* = 50 confocal micrographs of TMRM-stained monolayers, 25 per condition; r = 3 independent tumors from which monolayers were derived). ***P < 0.001; ****P < 0.0001 by an unpaired *t* test. Error bars represent the standard deviation. **(B and B′)** Assessment of cell death in Ad-Cre or Ad-Cre-null organoids subjected to the colony formation assay in Matrigel ±50 µM MitoTempo for 4 days, then fixed and stained for CC3 and DAPI. **(B′)** Ratio of CC3 intensity to DAPI intensity is reported (*n* = 20 confocal micrographs of individual organoids immunostained for CC3 and DAPI, r = 3 biological replicates). ****P < 0.0001 by one-way ANOVA. Scale bars 50 µm. Error bars represent the standard deviation. **(C)** Schematic of the in vivo metastasis assay. Images were created using https://BioRender.com/8c444de (images isolated for use in Illustrator during figure preparation). **(D)** 20-µm sections of lungs harvested 1 wk postinjection from NSG mice injected with clusters of MMTV-PyMT;Cdh1^fl/fl^;Cre^ERT2^; Rosa26-mTmG tumor cells subjected to various pretreatment conditions. Lungs are stained with DAPI, and metastases are detectable with tdTomato signal (E-cadherin intact) or eGFP (E-cadherin deleted). Scale bars, 200 µm. **(E)** Quantification of metastasis counts from the in vivo metastasis assay. Detectable metastases larger than 100 µm were counted, then divided by the total lung parenchymal area. Each datapoint represents an average of metastases detected in three nonconsecutive 20-µm lung tissue sections (*n* = 28 total mice; r = 2 independent donor tumors). **P < 0.01 by one-way ANOVA. Error bars represent the standard deviation. CTCF, corrected total cellular fluorescence; CC3, cleaved caspase-3.

**Figure S2.**
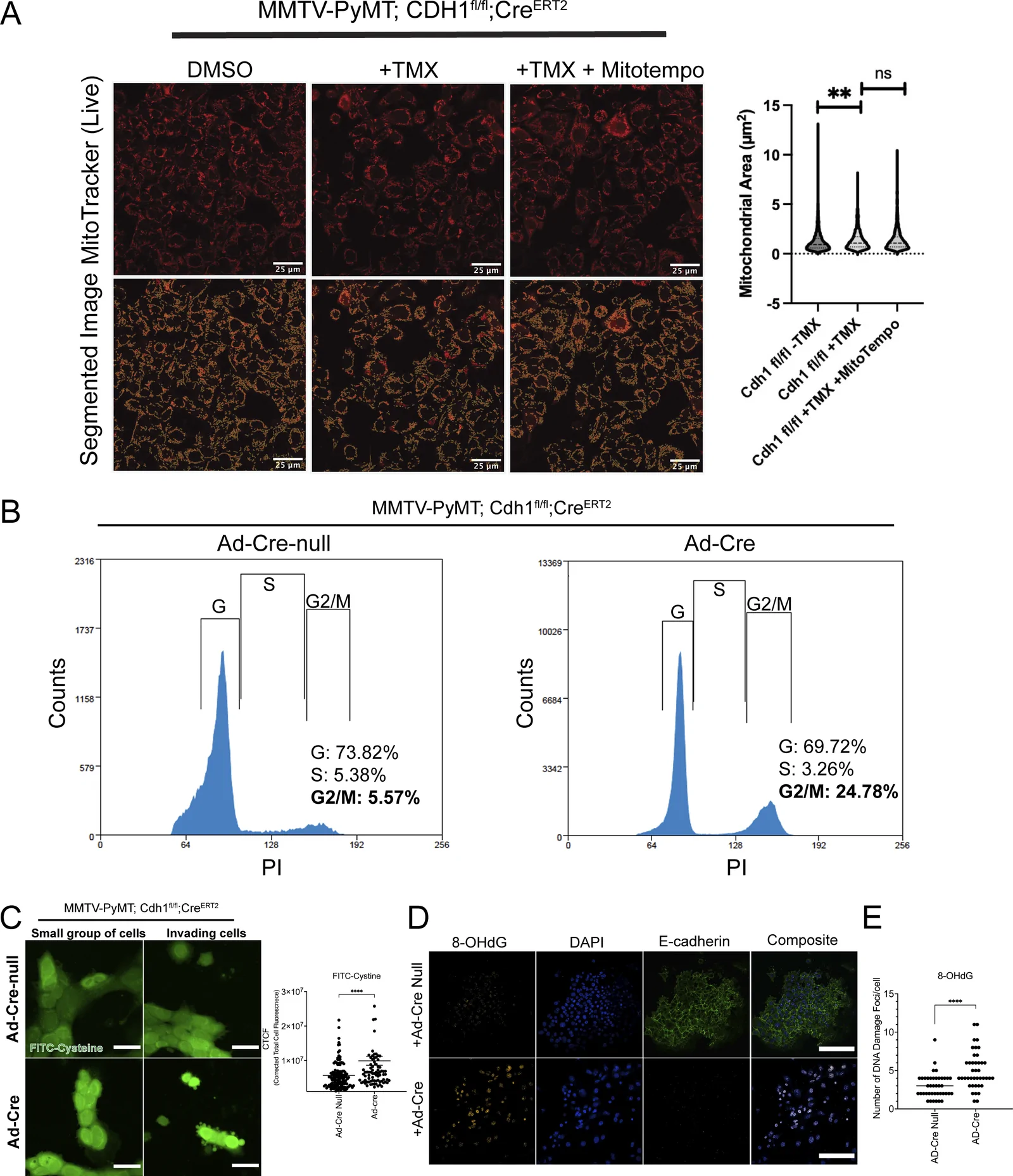
**Loss of E-cadherin increases mitochondrial size, G2/M accumulation, cysteine uptake, and oxidative DNA damage. (A)** Analysis of mitochondrial properties in MMTV-PyMT;Cdh1^fl/fl^;Cre^ERT2^; Rosa26-mTmG cells treated with DMSO or TMX. Segmented mitochondria are shown in the bottom panel. Quantification was carried out by automated segmentation of mitochondria stained with MitoTracker to measure area (*n* = 5,803 mitochondria, r = 3 biological replicates). P < 0.0001 by one-way ANOVA. **P < 0.01. **(B)** FACS histogram for control Ad-Cre-null (E-cadherin-intact control) and Ad-Cre (E-cadherin deleted) MMTV-PyMT;Cdh1^fl/fl^ cells assessed for cell cycle stage. **(C)** Assessment of FITC-cystine uptake in clusters of MMTV-PyMT; Cdh1^fl/fl^ cells treated with Ad-Cre or Ad-Cre-null. Scale bar, 20 µm. Fluorescence was assessed using CTCF (*n* = 218 clusters, r = 3 biological replicates). P < 0.0001 by Mann–Whitney U test. ****P < 0.0001. **(D)** Confocal micrographs (40×) of 8-OHdG adducts identified in Ad-Cre-null and Ad-Cre–treated tumor cell clusters. Counterstains included are DAPI and E-cadherin (ECCD-2 clone). **(E)** Quantification of the number of 8-OHdG-positive foci per cell in the clusters shown in D (*n* = 41 clusters, r = 3 biological replicates). P < 0.0001 by an unpaired *t* test. ****P < 0.0001. CTCF, corrected total cellular fluorescence.

We therefore hypothesized that the apoptosis-inducing surge of ROS following E-cadherin deletion was due to mitochondrial ROS production. To validate this concept, we used fluorescent biosensors and antibodies to measure mitochondrial superoxide (MitoSOX), mitochondrial membrane potential (tetramethylrhodamine methyl ester, TMRM), cell cycle status, FITC-cysteine uptake, and apoptosis levels in Cdh1-intact and Cdh1-deleted cells. Mitochondrial ROS and TMRM-reported mitochondrial potential were both significantly increased following loss of E-cadherin (Fig. 2, A–A″), providing direct evidence that E-cadherin protein levels affect mitochondrial oxidative state and function. Interestingly, we observed a significant increase in mitochondrial size in Cdh1-deleted cells, when compared to control, which was not rescued by MitoTempo treatment, as assessed by MitoTracker staining (Fig. S2 A). This result aligns with our finding that deletion of E-cadherin is associated with a significant decrease in the abundance of the Drp1 receptors Mid51/Mief1 and Mfn2, which regulate mitochondrial fission and fusion (Kamerkar et al., 2025). In addition, we observed that an increased proportion of Cdh1-deleted cells were arrested in G2/M by FACS cell cycle analysis (Fig. S2 B). Increased uptake of FITC-cystine by Cdh1-deleted cells suggests increased substrate uptake for glutathione synthesis and ROS detoxification (Fig. S2 C). The G2/M arrest could be explained by an increase in DNA damage that we observed following Cdh1 deletion, assayed as 8-OHdG staining (Fig. S2, D and E). Notably, treatment with MitoTempo reduces cleaved caspase-3 levels in Cdh1-deleted cells to the level of Cdh1-intact controls (Fig. 2, B and B′), providing functional evidence that mitochondrial ROS drives apoptosis following Cdh1 deletion.

We next sought to test whether scavenging of mitochondrial ROS with MitoTempo could rescue colony formation in vivo in the tail-vein experimental metastasis assay. Prior work has shown that MitoTempo accumulates in mitochondria at concentrations several orders of magnitude higher than the cytosol and could therefore persist significantly in vivo (Murphy and Smith, 2007). We hypothesized that preloading cells with MitoTempo prior to injection could reduce mitochondrial ROS levels and restore metastasis of Cdh1-deleted cells. To test this hypothesis, we harvested mammary tumor organoids from MMTV-PyMT;Cdh1^fl/fl^;Rosa26-mTmG;Cre^ERT2^ tumors, treated them with DMSO control or tamoxifen for 48 h to induce Cdh1 recombination, and confirmed recombination by the switch from red-to-green fluorescent protein expression. Organoids were then exposed to either 50 μM MitoTempo or DMSO control for an additional 7 days in suspension. Organoids were then digested to clusters of 4–10 cells and introduced into immunodeficient NOD/SCID/γ chain (NSG)–deleted mice by tail-vein injection. At 1 wk postinjection, lungs were collected and subsequently sectioned and imaged for analysis of metastatic burden (schematized in Fig. 2 C).

Control mice injected with Cdh1-intact cancer cells showed similar levels of metastatic burden in DMSO- vs. MitoTempo-treated conditions. In contrast, experimental mice injected with Cdh1-deleted cancer cells that were pretreated with MitoTempo showed a significant increase in metastatic capacity compared with their DMSO-treated counterparts (Fig. 2, D and E). Elevated mitochondrial ROS therefore contributes to the reduced metastatic efficiency observed following E-cadherin loss. Taken together, our results reveal that E-cadherin plays a critical role in limiting both mitochondrial ROS and mitochondrial membrane potential in disseminating cancer cells. Treatment with a mitochondrial ROS scavenger, MitoTempo, was sufficient to rescue cell survival and colony formation in vitro and metastasis formation in vivo. Our observation of an inverse correlation between E-cadherin levels and mitochondrial membrane potential is consistent with conclusions in a recent study done in breast cancer cell lines (Begum et al., 2021).

**Figure 3.**
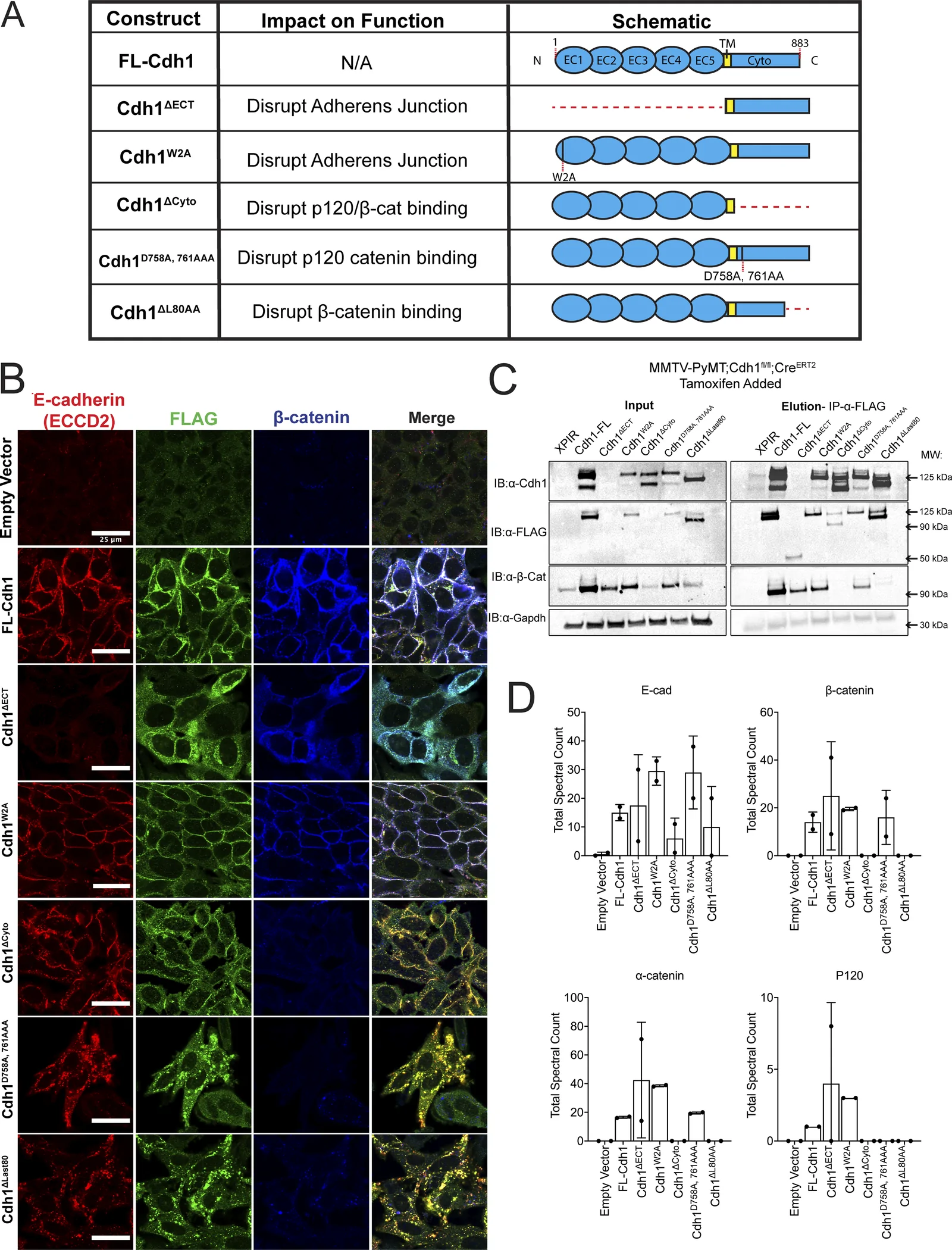
**Design and construction of engineered E-cadherin constructs to identify domain-specific functions in ROS generation. (A)** Table of E-cadherin constructs generated and their expected biological function. **(B)** Monolayers derived from MMTV-PyMT;Cdh1^fl/fl^;Cre^ERT2^; Rosa26-mTmG tumor organoids overexpressing, via lentiviral transduction, each of the E-cadherin constructs. Cells were fixed and imaged at ∼60–70% confluence. Fixed monolayers were stained with E-cadherin (ECCD-2 clone, extracellular domain), FLAG (overexpression constructs), and β-catenin. Scale bars, 25 µm. **(C)** FLAG immunoprecipitation of overexpressed E-cadherin constructs from MMTV-PyMT;Cdh1^fl/fl^;Cre^ERT2^ monolayers. Whole lysate is shown on the left, and IP elution is shown on the right. Lysates were immunoblotted for E-cadherin (ECCD2 clone), FLAG, β-catenin, and Gapdh. **(D)** Spectral counts identified for each assessed binding partner of E-cadherin by AP-MS/MS. Dots represent spectral counts per protein for each biological replicate (r = 2 independent donor tumors). Error bars represent the standard deviation. Source data are available for this figure: SourceData F3.

We next sought to isolate the specific domains and amino acids in E-cadherin that are critical for regulating intracellular ROS pathways. To achieve this goal, we developed Cdh1 constructs featuring various truncations and point mutations that are known to regulate adhesion and catenin binding. These constructs allowed us to test the roles of specific functional domains of E-cadherin in regulating ROS and cell survival in cancer cells in vitro and in vivo (Fig. 3 A). We first used western blotting to assess the expression of each rescue construct (Fig. S1 B). We then sought to distinguish whether the observed survival function of E-cadherin is due to extracellular adhesion, intracellular signaling, or a combination of the two. Constructs were introduced into MMTV-PyMT organoids, after which construct-expressing cells were selected for with puromycin and endogenous E-cadherin was deleted by tamoxifen treatment. Cells were subsequently subjected to immunofluorescence, western blotting, and immunoprecipitation to evaluate subcellular localization, protein abundance, and interactions with canonical binding partners (Fig. 3, B–D). Full length and Cdh1-W2A were both predominantly localized to the plasma membrane, while other constructs showed predominantly cytoplasmic localization (Fig. 3 B). Immunoprecipitation for the C-terminal FLAG tag revealed the expected patterns of binding to β-catenin and p120-catenin and expected molecular weights for each construct by western blot and the correct binding partners by mass spectrometry (Fig. 3, C and D).

**Figure 4.**
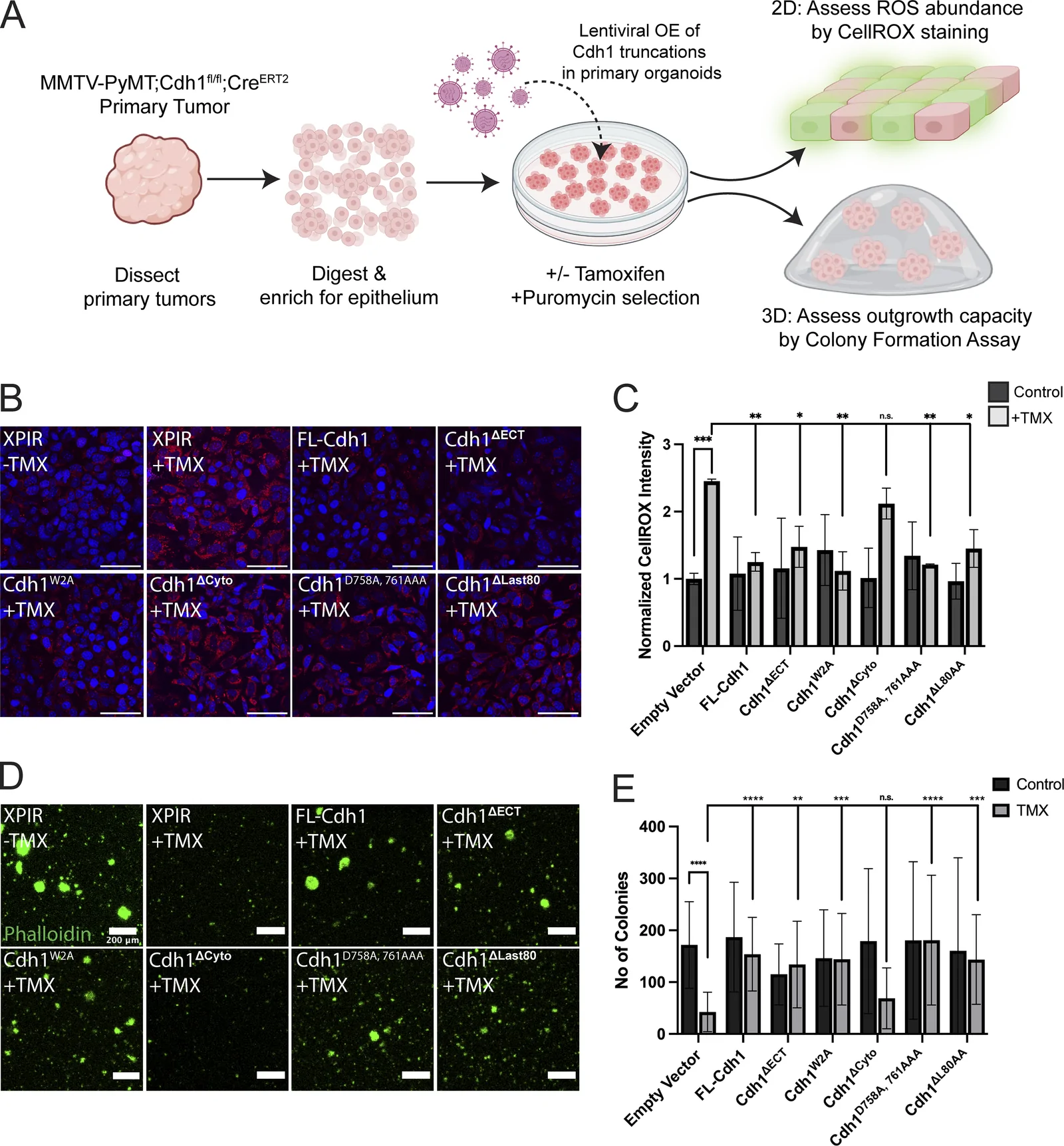
**Re-expression of E-cadherin is sufficient to reduce ROS and promote outgrowth in Cdh1-null cells. (A)** Schematic overview of the experimental approach to assess the functional contribution of E-cadherin truncation constructs to redox regulation and metastatic outgrowth potential. Primary mammary tumors from MMTV-PyMT;Cdh1^fl/fl^;Cre^ERT2^ mice were dissected, digested, and enriched for epithelial cells. Resulting primary organoids were transduced with lentiviral constructs encoding various E-cadherin truncations, followed by puromycin selection ± tamoxifen treatment to induce endogenous Cdh1 deletion. Functional assays were then performed to assess (1) ROS abundance using CellROX Green staining in 2D cultures and (2) outgrowth capacity using the colony formation assay in 3D Matrigel culture. **(B)** Assessment of ROS abundance in monolayers of MMTV-PyMT;Cdh1^fl/fl^;Cre^ERT2^ cells expressing each E-cadherin construct. Live cells stained with CellROX Green and DAPI were imaged by scanning confocal microscopy (40×). Scale bars, 50 µm. **(C)** Quantification of ROS abundance in MMTV-PyMT;Cdh1^fl/fl^;Cre^ERT2^ monolayers expressing E-cadherin constructs using CellROX intensity normalized to DAPI intensity (*n* = 28 wells, r = 2 biological replicates). Interaction, *P < 0.05 by 2-way ANOVA with multiple comparisons. Treatment (Control vs. TMX) P <0.001. *P < 0.05, **P < 0.01, ***P < 0.001. Error bars represent the standard deviation. **(D)** Organoids overexpressing E-cadherin constructs were subjected to the colony formation assay following treatment with DMSO or TMX. Images were taken by scanning confocal microscopy (10×). Scale bars, 200 µm. **(E)** Quantification of colony counts for each overexpression condition (*n* = 450 total wells, r = 3 biological replicates). Interaction, P < 0.0001; TMX treatment, P < 0.0001 by two-way ANOVA with multiple comparisons. ****P < 0.0001, ***P < 0.001, **P < 0.01. Error bars represent the standard deviation.

We next assessed the capacity of different domains of E-cadherin to rescue the elevated ROS and reduced survival phenotypes observed following CDH1 deletion (Fig. 4 A). CellROX fluorescence was compared between monolayers of MMTV-PyMT cells stably expressing each construct in both Cdh1-deleted (+TMX) and vehicle control (DMSO) conditions. Cdh1^ΔCyto^ was unable to reduce cellular ROS levels significantly following Cdh1 deletion, while all other constructs rescued ROS to baseline, including Cdh1^ΔEct^ (Fig. 4, B and C). Cdh1^ΔCyto^ was also unable to rescue colony formation significantly following Cdh1 deletion, while all other constructs restored colony formation to baseline, including Cdh1^ΔEct^ (Fig. 4, D and E). We note that the W2A point mutation that abolishes adhesion had indistinguishable effects on colony formation as the Cdh1^ΔEct^ deletion, providing complementary evidence that Cdh1’s role in mediating intercellular adhesion is not essential to its role in survival in this context. Taken together, our data reveal that the extracellular domains and catenin binding sites of Cdh1 are dispensable for ROS reduction and cell survival in colony formation assays in vitro. Instead, it is the cytoplasmic domain of Cdh1 that is essential.

**Figure 5.**
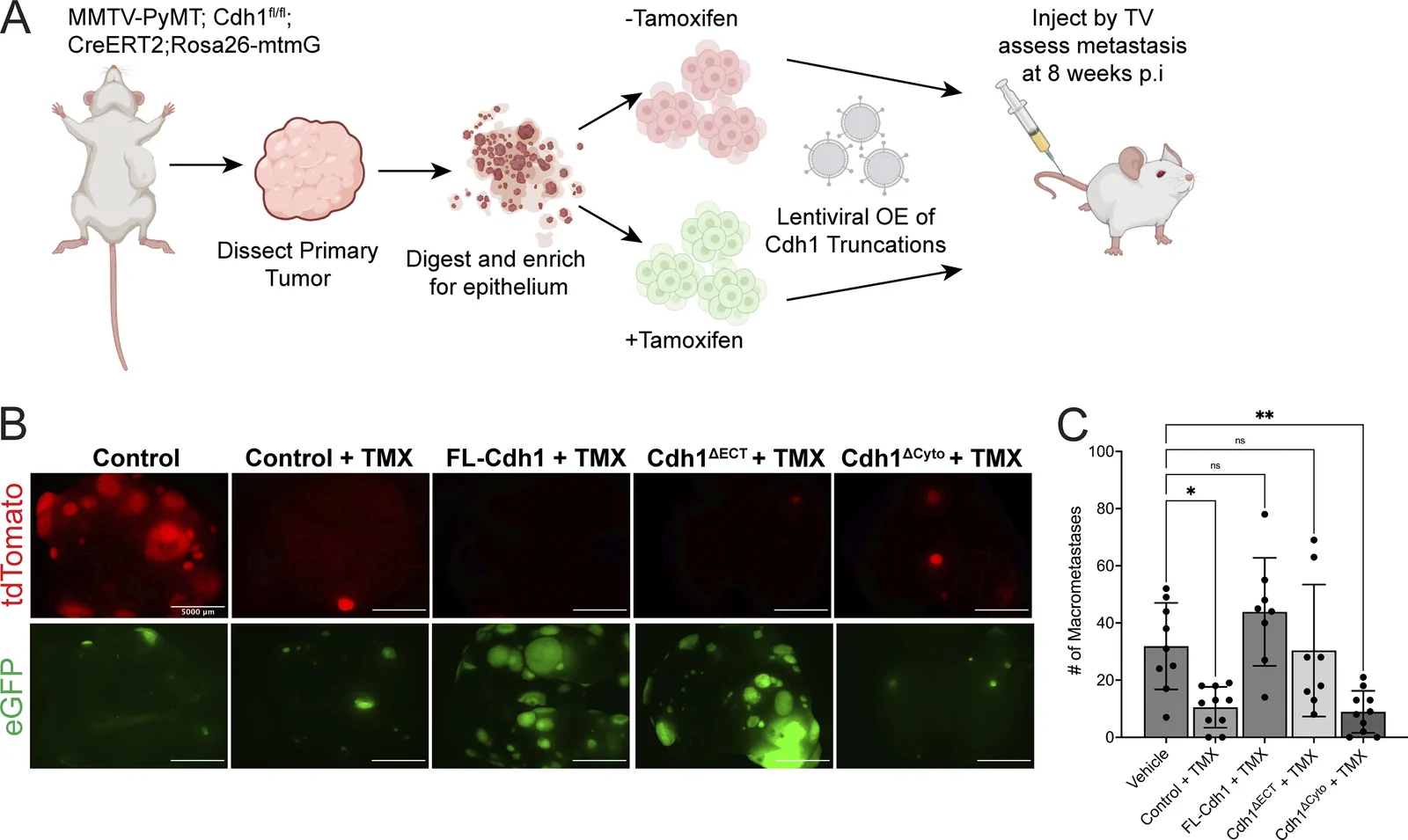
**Extracellular domain of E-cadherin is dispensable for metastatic outgrowth in vivo. (A)** Schematic of the in vivo tail-vein metastasis assay. Images were generated using https://BioRender.com/8c444de (images isolated for use in Illustrator during figure preparation). **(B)** Fluorescent stereomicroscopy images of lungs from NSG mice injected with MMTV-PyMT;Cdh1^fl/fl^;Cre^ERT2^; Rosa26-mTmG tumor cells dissected 8 wk postinjection. The presence of green signal is a direct reporter for recombination at the mTmG locus and an indirect reporter for Cdh1 deletion. These panels are reproduced in Fig. S3 A to provide greater context. **(C)** Quantification of metastasis counts in B. Statistical analysis conducted with one-way ANOVA with multiple comparisons (*n* = 45 mice, r = 3 biological replicates). ****P < 0.0001. Error bars represent the standard deviation.

**Figure S3.**
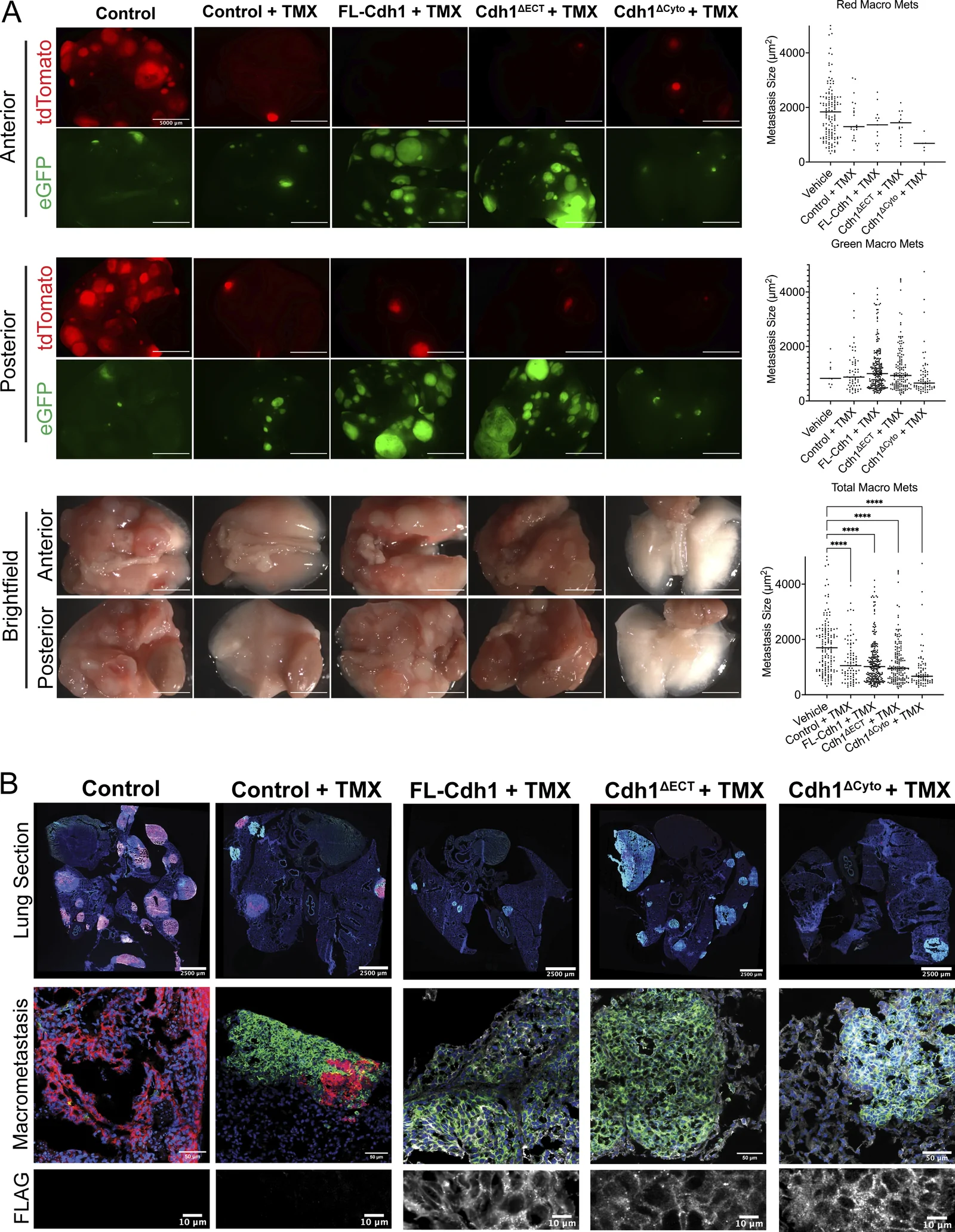
**Characterization of lung macrometastases and localization of E-cadherin rescue constructs. (A)** Representative stereomicroscope images (tdTomato, eGFP, and bright-field channels) of lungs harvested from NSG mice injected via tail vein with organoid-derived epithelial cells transduced with the indicated E-cadherin constructs and treated with tamoxifen to delete endogenous Cdh1. Fluorescent labeling enables visualization of macrometastases derived from tdTomato+ (red) and eGFP+ (green) cells from anterior and posterior lung surfaces. Right panels show the sizes of tdTomato+, eGFP+, and total macrometastases detected by stereomicroscopy. *n* = 45 mice across three independent donor tumors. Each point represents one macrometastasis; horizontal lines indicate mean. ****P < 0.0001 by one-way ANOVA. Some of these panels are reproduced from Fig. 5 B to provide greater context. **(B)** 20-µm frozen lung sections derived from the lungs shown in A stained with anti-FLAG antibody to assess construct localization within macrometastatic lesions. Top row: whole-lung tiled scans. Middle: high-magnification images of representative macrometastases in each condition. Bottom: FLAG signal (white) shows construct subcellular localization within individual macrometastatic lesions.

To validate these conclusions in vivo, we transduced cells with empty vector or constructs encoding FL-Cdh1, Cdh1^ΔEct^, or Cdh1^ΔCyto^ in MMTV-PyMT cancer cells and subsequently carried out puromycin selection and tamoxifen-mediated recombination to induce Cdh1 deletion. These cells were then digested to clusters and injected into the tail vein of 8-wk-old female NSG mice (Fig. 5 A). At 8 wk postinjection, lungs were analyzed by stereomicroscopy for macrometastases (Fig. S3, A and B). The metastatic burden in each condition recapitulated the results of our in vitro colony formation assay. Positive control clusters with intact Cdh1 developed macrometastases at a higher rate than their tamoxifen-treated Cdh1-deleted counterparts (Fig. 5 B). The expression of either the FL-Cdh1 or the Cdh1^ΔECT^ constructs was able to rescue the ability of Cdh1-deleted cells to form metastases, whereas the Cdh1^ΔCyto^ construct was not (Fig. 5, B and C).

Collectively, our data reveal that the ability of Cdh1 constructs to rescue colony formation in Cdh1-deleted IDC cells depended specifically on the properties of their cytoplasmic domain. Cdh1 truncation constructs that abolished adhesion (W2A), deleted the extracellular domains of E-cadherin, or eliminated β- or p120-catenin binding were able to rescue colony formation, and Cdh1^ΔEct^ rescued metastasis formation in the lungs. Conversely, an E-cadherin construct lacking the entire cytoplasmic domain was unable to rescue colony formation in vitro or metastasis formation in vivo. Critically, rescue of colony formation by E-cadherin mutant constructs correlated with their ability to suppress mitochondrial ROS, further substantiating the link between mitochondrial ROS and E-cadherin–mediated cancer cell survival. Taken together, our data support an adhesion- and catenin-independent role of Cdh1 in suppressing mitochondrial ROS and promoting cell survival during metastasis of invasive ductal breast cancer cells.

E-cadherin is conventionally viewed as an invasion and metastasis suppressor, whose ablation leads to a loss of cell–cell adhesion and acquisition of a migratory, invasive EMT phenotype (Berx and Van Roy, 2001; Christofori and Semb, 1999; Takeichi, 1993). This concept is well supported by clinical data and experimental animal models of invasive lobular breast cancer and diffuse gastric cancer, both of which frequently involve loss of Cdh1 during progression (Derksen et al., 2006). However, loss of Cdh1 is not sufficient for tumor formation; Cdh1^−/−^ mammary epithelial cells can invade past the basement membrane but do not disseminate widely into the stroma (Schipper et al., 2019; Shamir et al., 2014). Analyses of clinical IDC samples revealed that E-cadherin abundance is maintained in metastases relative to paired primary breast tumors, suggesting that disseminated cells gain a survival advantage by retaining or re-expressing E-cadherin (Chao et al., 2010; Chao et al., 2012; Kowalski et al., 2003). E-cadherin expression appears dynamic even within metastases, as it has been observed in patient samples to be high in micrometastases and low again in large growing metastases (Chao et al., 2010).

Across these studies, E-cadherin’s role in cancer progression has been viewed through an adhesion-centric lens, with adhesive cell–cell interactions promoting or suppressing outgrowth depending on a cell’s context in the metastatic cascade. The major conclusion of our study is that the domains responsible for homotypic binding—the extracellular EC repeats—are dispensable for survival during colony formation *ex vivo* and experimental metastasis to the lungs *in vivo*. E-cadherin proteins lacking the extracellular domain or harboring the W2A point mutation rescue colony formation in vitro. The extracellular domain deletion construct also promotes cell survival during hematogenous metastasis, despite its inability to support cell–cell adhesion.

Our findings identify a novel function of E-cadherin in metastasis, through which the intracellular domain mitigates mitochondrial oxidative stress, thereby promoting cell survival and increasing metastatic burden. E-cadherin’s complex dynamics at the plasma membrane, several routes of internalization, and regulatory complexity of trafficking (Bryant and Stow, 2004) yield many potential interactions that could regulate essential cellular functions, such as metabolism and signal transduction. Future studies could characterize the intracellular dynamics of E-cadherin, through the use of biosensors and proteomics, to assess how protein–protein interactions of E-cadherin are altered upon internalization and during cancer invasion. Such studies could also illuminate how E-cadherin dynamics support and regulate the distinct steps of metastasis in additional breast cancer subtypes and across solid tumor types.

## Materials and methods

### Isolation and 3D culture of primary mammary tumor organoids

Mammary tumors were collected from 10- to 12-wk-old MMTV-PyMT mice, mechanically disrupted with a scalpel, and enzymatically digested on a shaker (180 rpm, 37°C, 60 min) in the following collagenase solution: DMEM/F12 (10565-018; Gibco), 2 mg ml^−1^ collagenase (C2139; Sigma-Aldrich), 2 mg ml^−1^ trypsin (27250-018; Gibco), 5% FBS (F0926; Sigma-Aldrich), 5 μg ml^−1^ insulin (I9278; Sigma-Aldrich), and 50 μg ml^−1^ gentamicin (15750; Gibco). Digested tumors were centrifuged at 400 × *g* for 10 min, then treated with 2 U μl^−1^ DNase (D4263; Sigma-Aldrich). This was followed by four differential centrifugations (400 × *g*, 3 s) to separate the epithelial cell clusters from stromal cells. Smaller cell clusters (100–200 cells each) were then enriched using differential centrifugation at a lower speed (150 *g*, 3 s). Organoids were then resuspended in organoid medium (DMEM/F12 (10565-018; Gibco), 1% insulin–transferrin–selenium (51500-056; Gibco), 1% penicillin–streptomycin (P4333; Sigma-Aldrich), and 2.4 nM FGF2 (F0291; Sigma-Aldrich)), then added to nonadherent plates for virus infection or tamoxifen treatment.

### Deletion of Cdh1 in mammary tumor organoids

Adenoviral delivery of Cre recombinase: Prior to embedding in Matrigel, freshly isolated MMTV-PyMT organoids were infected with Adeno-Cre (1045; Vector BioLabs) or Adeno-GFP (1060; Vector BioLabs) at a multiplicity of infection of ∼10^7^ viral particles per 1000 organoids. Infection was carried out in suspension culture, in 50 μl DMEM/F12 overnight at 37°C. 4-hydroxytamoxifen-induced Cre activation: Organoids isolated from MMTV-PyMT; Cdh1^fl/fl^; Cre^ERT2^ or MMTV-PyMT; Cdh1^fl/fl^; Cre^ERT2^; Rosa26-mTmG mice were treated with 50 nM of 4-hydroxytamoxifen (H7904; Sigma-Aldrich) for 48 h in suspension. Tamoxifen was washed out by a 10-min incubation with PBS, and organoids were subsequently cultured in FGF2-containing organoid medium.

### Colony formation assay and ROS inhibitor screen

Freshly isolated organoids were treated with tamoxifen for 24–48 h to induce Cdh1 deletion. Organoids were then washed twice using PBS without Ca^2+^ or Mg^2+^ and trypsinized into small cell clusters by incubating with 1X TrypLE for 10–15 min at 37°C. Cells were resuspended in PBS (without Ca^2+^ or Mg^2+^), filtered through a 40-μm filter, and counted using a hemocytometer. Cells were resuspended in 30-µl Matrigel and cultured in 96-well plates with FGF2-containing organoid medium, then supplemented with 50 nM of tamoxifen for 7 days. Independent genotype-validated mice were used for each biological replicate. The number of colonies formed in each well was measured using a high-content imager (ImageXpress Micro XLS Widefield High-Content Analysis System, Molecular Devices), and ImageJ (Schindelin et al., 2012) was used for quantification. Briefly, images were processed in batch mode to reduce noise and background, then thresholded based on fluorescent phalloidin staining using a fixed intensity threshold range. Adjustments were applied uniformly across the whole image field. Binary masks were then generated using a black background setting and subjected to watershed segmentation to separate adjacent or touching objects.

Segmented objects were quantified using ImageJ’s Analyze Particles function, selecting for objects with an area threshold range of 300–5,000 µm^2^ and circularity between 0.10 and 1.00. Input images were 4× magnification fluorescent images of individual wells, and each output count reflects the total number of colonies within a given well that fell within the defined size and circularity range. Processing was performed in batch mode to ensure consistent segmentation and quantification across all images.

Small molecule and protein reagents used in this study included the following: an apoptosis inhibitor (z-VAD-FMK), a TGFβR1 inhibitor (SB525334; S1476; SelleckChem), NAC (A7250; Sigma-Aldrich), a NOX 1,4 inhibitor (GKT831; S71771; SelleckChem), a nonselective NOX inhibitor (DPI; 0504; Tocris), a lipoxygenase inhibitor (NDGA; S3984; SelleckChem), a xanthine oxidase inhibitor (febuxostat; 4427; Tocris), a mitochondrial ROS inhibitor (MitoTempo; SML0737; Sigma-Aldrich). All drug treatments were added on the same day as embedding clusters in Matrigel.

### Protein isolation

Organoids grown in suspension were transferred to microfuge tubes and centrifuged at 400 × *g* for 10 min. Residual culture medium from the pellet was removed by washing twice in ice-cold PBS. Organoids were then resuspended in <50 μl of ice-cold lysis buffer, vortexed, and left on ice for 30 min. Lysis buffer was made fresh, using 142.8 μl of 7× protease inhibitor stock (two cOmplete Mini Tablets (11836153001; Sigma-Aldrich), 2 PhosphoSTOP tablets (4906837001; Sigma-Aldrich), and 2.856 ml dH2O), 100 μl RIPA buffer (20-188; Millipore), 50 μl glycerol (G5516; Sigma-Aldrich), 10 μl 10% SDS (L3771; Sigma-Aldrich), 697.2 μl of dH2O. The samples were centrifuged for 10 min at 18,000 *g* (4°C). The supernatants were then transferred into new, cold Eppendorf tubes and stored at −80°C.

### Western blotting

Whole-cell protein lysates were thawed on ice for 30 min before use. The amount of protein in each sample was quantified using a standard BCA assay kit (23225; Thermo Fisher Scientific). Equal protein amounts (20 µg) were loaded, with Laemmli sample buffer (1610747; Bio-Rad) and β-mercaptoethanol, in 4–15% Mini-PROTEAN precast gels (456-1084; Bio-Rad). SDS–PAGE was performed at 200 V for 30–40 min, or until the dye front had just run off the gel. Gels were transferred onto PVDF membranes (IPFL07820; Millipore) at 100 V for 1 h at 4°C. Membranes were then blocked with Odyssey blocking buffer (92750000; LI-COR) for 1 h at room temperature. Primary antibodies were diluted at 1:1,000 into a 1:1 mixture of blocking buffer and TBS-T (TBS + 0.2% Tween [P7949; Millipore]). Primary antibodies used in the study included rat E-cadherin (ECCD-2 clone, M108; Takara), rabbit β-actin (A2103; Sigma-Aldrich), mouse FLAG (F1804; Sigma-Aldrich), rabbit αE-catenin (3236; Cell Signaling), rabbit β-catenin (8480; Cell Signaling), E-cadherin (14472; Cell Signaling Technology), Drp1 (611113; BD Transduction Laboratories), phospho-Drp1 S616 (3455; Cell Signaling Technology), phospho-Drp1 S637 (6319; Cell Signaling Technology), Opa1 (612607; BD Transduction Laboratories), Mfn1 (13798-1-AP; Proteintech), Mfn2 (ab57602; Abcam), Mid51/Mief1 (20164-1-AP; Proteintech), Mid49/Mief2 (16413-1-AP; Proteintech), Mff (gifted from Dr. Alexander M. van der Bliek, University of California Los Angeles, Los Angeles, CA, USA; Gandre-Babbe and van der Bliek, 2008), Fis1 (10956-1-AP; Proteintech), Tom20 (11802-1-AP; Proteintech), α-tubulin (2125; Cell Signaling Technology), Hsp60 (12165; Cell Signaling Technology), Gapdh (MA5-15738; Thermo Fisher Scientific). Membranes were washed three times with 0.1% TBS-T and then incubated with secondary antibodies, diluted at 1:8,000, for 1 h at room temperature. Secondary antibodies used included anti-rat IRDye 800CW (925-32219; LI-COR), anti-mouse IRDye 680RD (925-68070; LI-COR), anti-rabbit IRDye 800CW (925-32211; LI-COR). Membranes were washed three times, for 5 min each, using 0.1% TBS-T. Membranes were scanned using the Odyssey CLx imaging system. Band intensities were quantified using ImageJ and normalized to loading control intensity (β-actin or Gapdh).

### Immunofluorescence

Colonies in Matrigel were fixed using 1% PFA with 0.25% glutaraldehyde for 10 min. They were then washed three times in PBS. Gels were permeabilized using 0.5% Triton X-100 in PBS (X100; Sigma-Aldrich) for 30 min at room temperature. Samples were blocked for 2 h with 10% FBS, 1% BSA in PBS at room temperature, incubated with primary antibodies diluted in 1% FBS, 1% BSA, 0.2% Triton X in PBS overnight at 4°C, then washed three times for 10 min each using PBS. Secondary antibodies were added, diluted in 1% FBS, 1% BSA, 0.2% Triton X in PBS and incubated for 3 h at room temperature. Samples were washed three times with PBS and stored at 4°C until imaging. Monolayers derived from MMTV-PyMT organoids overexpressing lentiviral constructs were fixed with 4% PFA for 10 min at room temperature followed by three PBS washes. Permeabilization was carried out with 0.5% Triton X-100 for 30 min. Cells were blocked with 10% FBS, 1% BSA in PBS for 2 h. Primary and secondary antibodies were diluted in 1% FBS, 1% BSA in PBS and incubated for 2 h. Following secondary antibody staining, monolayers were washed three times with PBS, then stored for imaging. Antibodies used for monolayer staining include E-cadherin (ECCD-2 clone, M108; Takara), FLAG (F1804; Sigma-Aldrich), β-catenin (8480; Cell Signaling).

Lungs from tail-vein experiments were dissected and flushed with PBS through the left ventricle to clear excess blood. Lungs were then fixed in 4% PFA for 24 h at 4°C, followed by dehydration in 15% sucrose for 24 h, then 30% sucrose for 24 h. Fixed lungs were then embedded into optimal cutting temperature (OCT) compound, and frozen by floating blocks on a liquid nitrogen bath. Sections (20-μm-thick) were cut onto Superfrost Plus Gold Microscope slides (15-188-48; Thermo Fisher Scientific) at −20°C using a cryostat. OCT was removed from slides by incubating in PBS for 1 h at room temperature. The sections were otherwise processed and stained identically to 3D gel–embedded organoids.

The number of independent biological replicates for each imaging experiment is specified in the corresponding figure legend. Primary antibodies used in the studies include rat E-cadherin (1:100, M108; Takara), 8-OHdG (sc-66036; Santa Cruz), rabbit β-catenin (1:200, 8480S; Cell Signaling), mouse anti-FLAG (1:200; F1814; Sigma-Aldrich). Nuclei were stained with DAPI (1:1,000, D571; Invitrogen). All secondary antibodies used were Alexa Fluor conjugates (1:200; Invitrogen). Samples were imaged using a laser-scanning confocal microscope equipped with a tunable DUX-VB detector (AXR; Nikon Instruments). Immunofluorescence images were gathered using a CFI60 Apochromat Lambda S LWD 40× water immersion objective lens (N.A. 1.15, W.D. 0.59–0.61 mm, FOV 22 mm, DIC, correction collar 0.15–0.19 mm).

### Cloning and production of lentivirus

The murine Cdh1 plasmid construct was purchased from OriGene (NM_009864). Cdh1 was subcloned into a lentiviral plasmid backbone based on a modified Puro-Cre vector (plasmid # 17408; Addgene, mCMV promoter and no Cre encoding region). Point mutations or truncations of indicated plasmid constructs were generated by PCR. The open reading frames of all DNA plasmids were verified by Sanger sequencing and have been uploaded to Dryad: https://doi.org/10.5061/dryad.r2280gbv6.

Lentiviral particles were produced using Gibco LV-MAX Lentiviral Production System (A35684; Thermo Fisher Scientific), which allows for lentivirus generation in suspension HEK293F cells (included in kit) under serum-free conditions. Gibco Viral Production Cells were thawed into LV-MAX Production Medium and cultured in vented 125-ml flasks on an orbital shaker at 37°C with 8% CO_2_ and >80% humidity. Cells were passaged every 3–4 days to maintain a density range between 3.5 × 10^6^ and 5.5 × 10^6^ cells/ml and were expanded until the appropriate density for transfection was reached.

For lentiviral production, cells were transfected at a final density of 4.0 × 10^6^ cells/ml using a 3:2 ratio of lentiviral packaging plasmid (LPM) to transfer plasmid (LTV), with a total of 2.5 μg DNA per mL of culture. Plasmids were combined with LV-MAX Transfection Reagent in Opti-MEM and incubated for 10 min at room temperature before addition to culture. LV-MAX Enhancer was added 5–6 h after transfection to enhance viral production. Lentiviral supernatants were harvested 48 h after transfection. Supernatants were isolated by centrifugation at 1,300 × *g* for 15 min before filtration through a 0.45-μm membrane.

To concentrate the lentiviral particles, supernatants were layered on a 20% (wt/vol) sucrose solution prepared in PBS and subjected to ultracentrifugation at 20,000 RPM for 2 h at 4°C using a Beckman Coulter SW28 rotor. Supernatants were then aspirated, and the viral pellet was resuspended in DMEM/F12. Viral aliquots were stored at −80°C.

### Lentiviral overexpression in organoids

Organoids were isolated as described above, and then, 1,000 organoids were plated in 50 μl in 96-well nonadherent plates. Lentiviral overexpression constructs at 20 multiplicity of infection were combined with 3 μl of ViroMag per well and were mixed up to a total volume of 200 μl and incubated at room temperature for 30 min. 200 μl of combined virus, ViroMag, and media was added to each well, and then, the plate was placed on a magnetic plate (MF10000; OZ Biosciences) for 90 min at 37°C. The plate was removed from the magnet and returned to the incubator. The next day, 125 μl of media was replaced with media containing FGF2. On day 2, 125 μl of media was replaced with media containing 5 μg ml^−1^ puromycin (A1113803; Gibco) and 50 nM tamoxifen. Organoids were left for 3 days and then used in downstream applications.

### Tail-vein injection: Experimental metastasis assay

Tamoxifen- and DMSO-treated MMTV-PyMT Cdh1^fl/fl^ organoids overexpressing E-cadherin constructs were trypsinized into small cell clusters by incubating with TrypLE (12604013; Thermo Fisher Scientific) for ∼10–15 min at 37°C. Cells were resuspended in DMEM/F12 at a concentration of 50,000 cells distributed among 4-10 cell clusters per 100 μl. Host NSG mice for these experiments were 6- to 8-wk-old females. Prior to injection, tail veins were dilated by applying a heat lamp for 1-2 min. Using a 28-gauge needle, 100 μl of cells was injected via the tail vein of the mouse. Lungs from these mice were collected at 8 wk (E-cad domain expression) from the date of injection and examined for macrometastases by stereomicroscopy. Lungs were subsequently sectioned and immunostained for FLAG and stained with DAPI for assessment of subcellular localization. For MitoTempo pretreatment tail-vein assays, the same procedure was performed, and equal numbers of cells were injected. However, the lungs of mice were collected 1 wk postinjection and analyzed for the presence of metastases by scanning individual sections. Prior to trypsinization, organoids were treated with tamoxifen or DMSO for 48 h and then with 50 μM MitoTempo for 1 wk.

### Counting macro- and micrometastases

After fixation, lungs were visualized with a SteREO Discovery.V12 dissecting microscope (Zeiss), and the numbers of visible eGFP+ and tdTomato+ foci on each lung (macrometastases) were counted manually on both anterior and posterior aspects of the lungs. For high-magnification imaging of lung tissue and metastases, lungs were fixed in 4% PFA and embedded by coating in OCT, then frozen using a floating weigh boat on liquid nitrogen. 20-µm sections were cut using a cryostat directly onto glass slides. For processing individual sections, OCT compound was removed with three cold PBS washes followed by an additional fixation step with 4% PFA. Sections were stained with anti-FLAG primary antibody (1:250; F1814; Sigma-Aldrich) and Alexa Fluor 647–conjugated secondary antibody (1:250; A32787; Thermo Fisher Scientific). Micrometastases were imaged with the Axio Scan.Z1 microscope (Zeiss) using Zen Blue 2.1 with the following configurations: Fluor 5×/0.27NA M27 objective for coarse focus mapping, Plan Apochromat 20×/0.80NA M27 objective for fine focus mapping, Hamamatsu Orca Flash 4.0 (fluorescence) camera for image capture, and Colibri 7 VIS-LED fluorescent light source. Metastases arising from Cdh1^+/+^ donor tissue were always E-cad^+^. Metastases that arose from Cdh1^fl/fl^ donor tissue and consisted exclusively of tdTomato^+^ cells were counted as E-cad^+^. Metastases that were all eGFP^+^ or that contained >50% eGFP^+^ cells were scored as E-cad^−^ for the purposes of quantification. Zen (Zeiss) and QuPath (Bankhead et al., 2017) were used to adjust brightness/contrast levels to maximize image clarity, reduce noise background, identify, and count metastases larger than 100 μm. Representative images were opened in ImageJ to place scale bars and crop insets.

### Live-cell imaging

Organoids were isolated and treated with Ad-Cre as described above. 24-well glass bottom plates were coated with a thin layer of neutralized, fibrillar rat-tail collagen I (354236; Corning). A 20 μl spot of collagen was placed in the middle of each well, and then, the pipette tip was used to evenly spread collagen in a circle around the well. Excess collagen was removed with the pipette tip, and the plate was incubated in a 37°C incubator for 20 min. Organoids were counted and seeded at 250 organoids per well in organoid media. Organoids were allowed to attach and spread on collagen for 48 h, and then, cell dyes were added.

The CellROX Deep Red and CellROX Green reagents, 5 μM (C10422, C10444; Life Technologies), were incubated for 30 min and used to measure oxidative stress within the cells. MitoSOX, 1 μM (M36008; Life Technologies), was incubated for 15 min and used to measure mitochondrial oxidative stress within cells. Cystine-FITC (SCT047; Sigma-Aldrich) uptake was measured by incubating cells with 5 μM for 30 min at 37°C. TMRM (I34361; Thermo Fisher Scientific) stock solution (100 μM) was diluted 1:1,000 in DMEM/F12 media and incubated with cells for 30 min at 37°C.

Confocal imaging was carried out on a Nikon AXR laser-scanning confocal microscope (Nikon NIS-Elements C) equipped with the following shown in Table 1.

**Table 1.** Nikon AXR confocal microscope specifications used in this study

Component	Specification
Objective turret	CFI Plan Apochromat Lambda 10× objective lens, 0.45 NA, 4.0 mm WD, FOV 25 mm, DIC, spring-loaded (MRD00105)
	CFI Plan Apochromat Lambda S LWD 20× water immersion objective lens, 0.95 NA, 0.95 mm WD, FOV 22 mm, DIC, correction collar 0.11–0.23 mm (MRD77200)
	CFI Plan Apochromat Lambda 20× objective lens, 0.75 NA, 1.0 mm WD, 25 mm FOV, DIC, spring-loaded (MRD00205)
	CFI Apochromat Lambda S LWD 40× water immersion objective lens, 1.15 NA, 0.59–0.61 mm WD, 22 mm FOV, DIC, correction collar 0.15–0.19 mm (MRD77410)
	CFI Plan Apochromat Lambda D 60× oil immersion objective, 1.42 NA, 0.15 mm WD, 25 mm FOV, DIC, spring-loaded (MRD71670)
Laser lines	405 nm (100-mW diode) • 488 nm (120-mW OPSL) • 561 nm (80-mW OPSL) • 640 nm (60-mW diode)
Detectors	Tunable DUX-VB Detector, tunable GaAsP detectors
Scan module	Resonant 8 kHz scanner, 2048 × 2048 px, 8× frame-averaging unless noted
Pinhole	1.0 Airy unit (unless otherwise noted)

### Cell cycle analysis with FACS

Organoids were isolated and treated with Ad-Cre or tamoxifen to delete Cdh1, as described above. Organoids were then harvested, centrifuged, and washed two times in PBS without Ca^2+^ or Mg^2+^. Organoids were then digested into single cells by incubating with 10X TrypLE for 10–15 min at 37°C. Digestion was stopped with DMEM/F12 containing 50% FBS. Cells were then pelleted and fixed in 70% EtOH for 30 min at 4°C. Cells were washed 2 times with PBS to remove EtOH. Cells were then resuspended in 500 μl of FxCycle PI/RNase Staining Solution (F10797; Invitrogen). Samples were assessed on a Sony SH800S cell sorter equipped with 405-, 488-, and 561-nm solid-state lasers. A disposable 100-µm sorting chip (#LE-C3210; Sony) was used to sort cells. Chip calibration was performed automatically with the SH800 built-in Auto Setup beads (#LE-B3001; Sony) immediately before each experiment. FSC gain, SSC gain, and fluorescence detectors were used at manufacturer-recommended starting voltages, and tuned so that PI-negative cells were centered at ∼10^1^ on a log scale. The event rate was maintained below 1,000 events per second to maintain droplet stability. FSC-A vs. SSC-A was used to remove debris, and FSC-H vs SSC-H was used for isolation of single cells. Cell cycle phase distribution (G0/G1, S, and G2/M) was determined based on DNA content measured by PI fluorescence. Histogram plots and cell cycle modeling were performed using FlowJo v10 (BD Biosciences).

### Immunoprecipitation–mass spectrometry

Organoids were isolated, infected with lentivirus, and treated with tamoxifen as described above. On day 5 of infection, organoids were lysed using Pierce IP lysis buffer (87787; Thermo Fisher Scientific) containing 1X phosphatase and protease inhibitor described above. Organoids from 24 wells of a 96-well plate were lysed in 1 ml of lysis buffer. Lysed cells were incubated on ice for 30 min and centrifuged at 20,000 × *g* for 15 min. The supernatant was removed, and the BCA assay was performed as described above. For immunoprecipitation from primary organoids, 80 μl of prewashed anti-FLAG beads (M8823; Sigma-Aldrich) was added to a microfuge tube containing 200 μg of protein. Protein samples were incubated with magnetic beads overnight at 4°C in an end-over-end rotator. The next day, the tube was placed on a magnet and unbound protein was removed with four ice-cold PBS washes. Proteins were eluted from beads with 3× Flag peptide (F4799; Sigma-Aldrich) at 5 μg/μl in 125 μl. Protein elution concentration was determined by the BCA assay, and then, samples were submitted for LC-MS analysis.

Briefly, peptides were separated using an EASY-nLC 1200 system coupled to a timsTOF fleX mass spectrometer. Peptides were first loaded onto a 3-cm PepMap 100 C18 trapping column (Thermo Fisher Scientific), and then, bound peptides were washed with 12 μl of buffer A (0.1 % formic acid in LC-MS-grade water) coupled inline to a PepSep 15 cm × 75 µm ReproSil C18 analytical column (Bruker Daltonics). Peptides were eluted using a 30-min LC gradient at 500 nl/min beginning at 12% buffer B (0.1% formic acid in 80:20 LC-MS-grade acetonitrile:water) to 90% buffer B in 25 min before returning to baseline conditions. The TIMSTOF Flex was operated in data-dependent acquisition (PASEF) mode, in which all peptides from 0.6 – 1.6 1/k0 and m/z 100-1700 were observed in MS1 using a ramp time of 100 ms. A custom polygon was used to select ions for fragmentation that were most likely to have 2–4 charges and a m/z of 350–1700 with a target value of 50,000 ions and a minimum threshold of 5,000 ions for each fragmentation scan event. All other parameters were default for the PASEF mode in Compass 4.0. Resulting output files were analyzed in FragPipe 17 (Yu et al., 2020) using the UniProt/SwissProt reviewed sequences for the appropriate species. IonQuant was used to generate relative abundance for each respective file for downstream interpretation.

### Corrected total cell fluorescence quantification

Corrected total cell fluorescence = Integrated Density – (Area of selected cell × Mean fluorescence of background readings). These measurements were done using ImageJ. In short, cells from each image were outlined in ImageJ and measured for fluorescence intensity and shape descriptors. A portion of the image with no fluorescence was used to measure background fluorescence.

### Mice

All mouse husbandry, procedures performed, and assay endpoints were in accordance with protocols approved by the Johns Hopkins Medical Institute Animal Care and Use Committee (ACUC). All mice were female. The mammary tumor donor strains were backcrossed and maintained on the FVB/n background. MMTV-PyMT, mTmG, Cdh1fl/fl mice were obtained from the Jackson Laboratories. The R26::Cre-ERT2 mouse line was a gift from J. Nathans (Johns Hopkins University, Baltimore, MD, USA). The NSG mice used for tail-vein metastasis assays used in this study were obtained from Jackson Laboratories (RRID:IMSR_JAX:005557). In all experiments involving tumor mice, mice were euthanized when the tumor burden was 20 mm or less, consistent with our ACUC-approved protocol limit.

### Schematics

Schematics were designed in a licensed copy of BioRender unless otherwise noted.

### Western blots for mitochondrial proteins

Proteins were separated by SDS–PAGE and transferred onto Immobilon-FL Transfer membrane (Millipore). The membranes were blocked in PBS-T (PBS containing 0.05% Tween 20) containing 3% BSA at room temperature for 1 h and then incubated with primary antibodies in PBS-T containing 3% BSA at 4°C overnight. The membranes were washed three times in PBS-T, followed by incubation with appropriate secondary antibodies at room temperature for 1 h. After washing the membranes three times in PBS-T, fluorescence signals were detected using a Typhoon laser-scanner platform (Amersham). Proteins were detected using the following primary antibodies: E-cadherin (#14472; Cell Signaling Technology), Drp1 (#611113; BD Transduction Laboratories), phospho-Drp1 (Ser616, #3455; Cell Signaling Technology), phospho-Drp1 (Ser637, #6319; Cell Signaling Technology), Opa1 (#612607; BD Transduction Laboratories), Mfn1 (#13798-1-AP; Proteintech), Mfn2 (#ab57602; Abcam), Mid51/Mief1 (#20164-1-AP; Proteintech), Mid49/Mief2 (#16413-1-AP; Proteintech), Mff (gifted from Dr. Alexander M. van der Bliek, University of California Los Angeles, Los Angeles, CA, USA; Gandre-Babbe and van der Bliek, 2008), Fis1 (#10956-1-AP; Proteintech), Tom20 (#11802-1-AP; Proteintech), α-tubulin (#2125; Cell Signaling Technology), Hsp60 (#12165; Cell Signaling Technology), and Gapdh (#MA5-15738; Thermo Fisher Scientific).

### Mitochondrial quantification

Confocal analysis of mitochondrial properties was carried out with the Mitochondria Analyzer Pipeline (Chaudhry et al., 2020). In short, MitoTracker-stained mitochondria were imaged using a laser-scanning confocal microscope equipped with a CFI60 Apochromat Lambda S LWD 60× Oil immersion objective lens. The preprocessing workflow in ImageJ began with “Subtract Background” (using the rolling ball algorithm to smooth while preserving edges), followed by “Sigma Filter Plus” to reduce noise and “Gamma Correction” to nonlinearly remap brightness and amplify faint signals. All corrections were applied to the entire field of view and consistently across images in the same analysis. Once preprocessed, each image was binarized via the weighted mean (adaptive threshold) method and any remaining speckle noise was cleared using “Despeckle” and “Remove Outliers.” The clean binary masks were then converted into skeletons with “Skeletonize (2D/3D).” For particle analysis, mitochondrial metrics (count, area, perimeter, form factor, and aspect ratio) were measured directly from the 2D binary images using “Analyze Particles.”

### Statistical analysis

All data are presented as the mean ± standard deviation (SD) unless otherwise indicated. All experiments were performed in at least three independent biological replicates, unless stated otherwise. A P value <0.05 was considered statistically significant. Data were analyzed using GraphPad Prism 10. No statistical methods were used to predetermine sample sizes, but sample sizes are consistent with standards in the field. Mice were randomly assigned to treatment groups, and analyses of metastatic burden were performed in a blinded fashion. Throughout this study, biological replicates refer to tumors harvested from individual mice, whereas n denotes nested measurements within each of those biological replicates, unless otherwise noted.

### Online supplemental material

Fig. S1 shows western blot confirmation of E-cadherin KO and expression of constructs, impact of ROS inhibitors on colony formation, effect of E-cadherin loss on CellROX fluorescence in 2D, and western blot of mitochondrial dynamics proteins. Fig. S2 shows mitochondrial size, cell cycle analysis, and 8-OHdG staining in Cdh1-intact and Cdh1-deleted cells. Fig. S3 shows stereomicroscopy images of lung macrometastases.

## Supplementary Material

SourceData F3 is the source file for Fig. 3.

SourceData FS1 is the source file for Fig. S1.

## Data Availability

Data are available upon reasonable request. Sequence data for the E-cadherin mutations are available at https://doi.org/10.5061/dryad.r2280gbv6.
