# Supplementary material for: Nonadhesive functions of E-cadherin promote cancer cell survival and metastasis
Source: J Cell Biol. 2026 Oct 22;225(11):e202507222. doi: 10.1083/jcb.202507222 (PMC13689684; doi:10.1083/jcb.202507222)

Source Data for Figure 3C

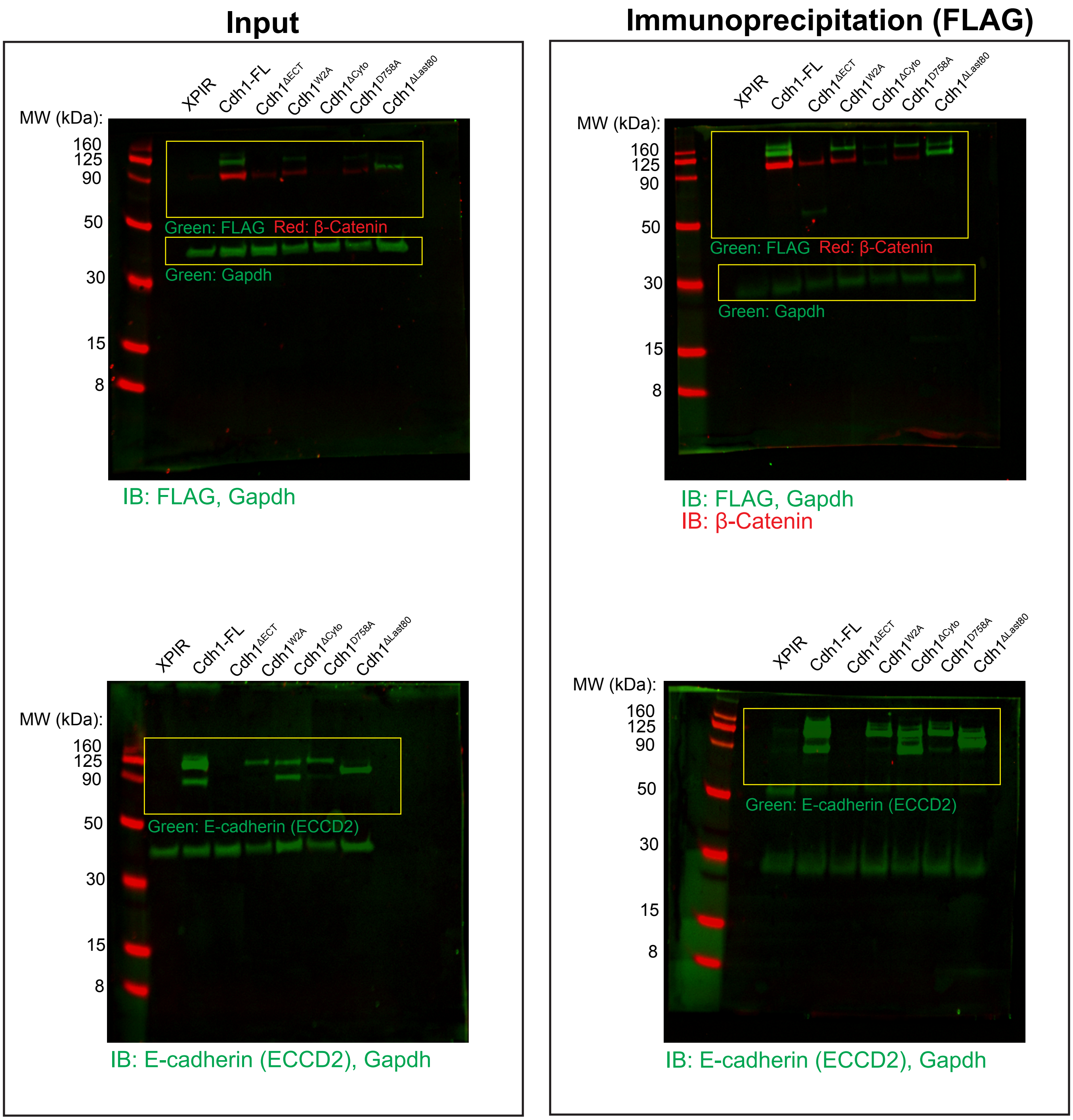

Supplement: Supplementary file 1 [file jcb_202507222_sourcedataf3.pdf]
