# Supplementary material for: Nonadhesive functions of E-cadherin promote cancer cell survival and metastasis
Source: J Cell Biol. 2026 Oct 22;225(11):e202507222. doi: 10.1083/jcb.202507222 (PMC13689684; doi:10.1083/jcb.202507222)

Supplementary Material: Uncropped Western Blots

WB for figure 3C: Immunoprecipitation of E-cadherin overexpresison constructs

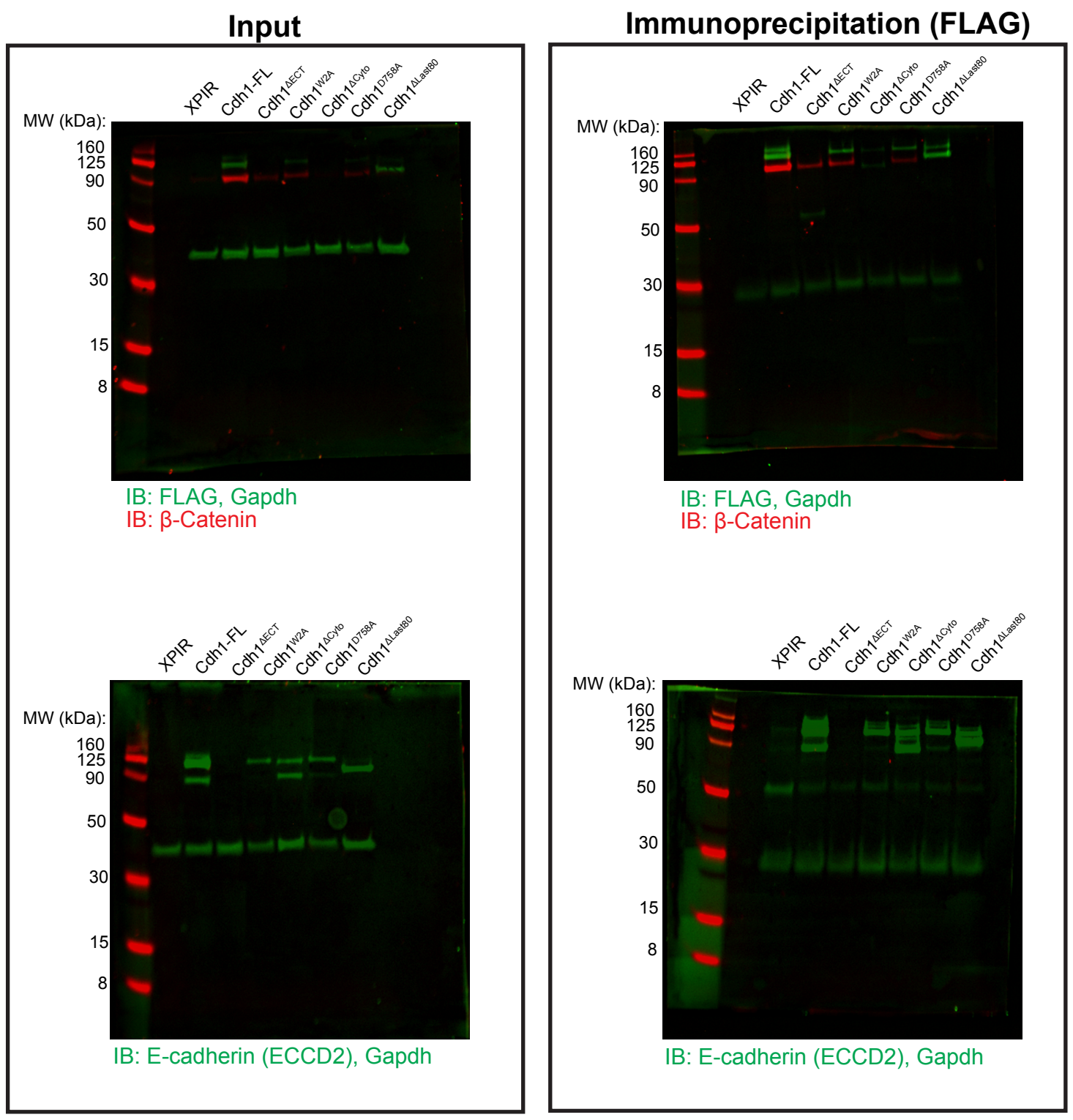

WB for figure S1A

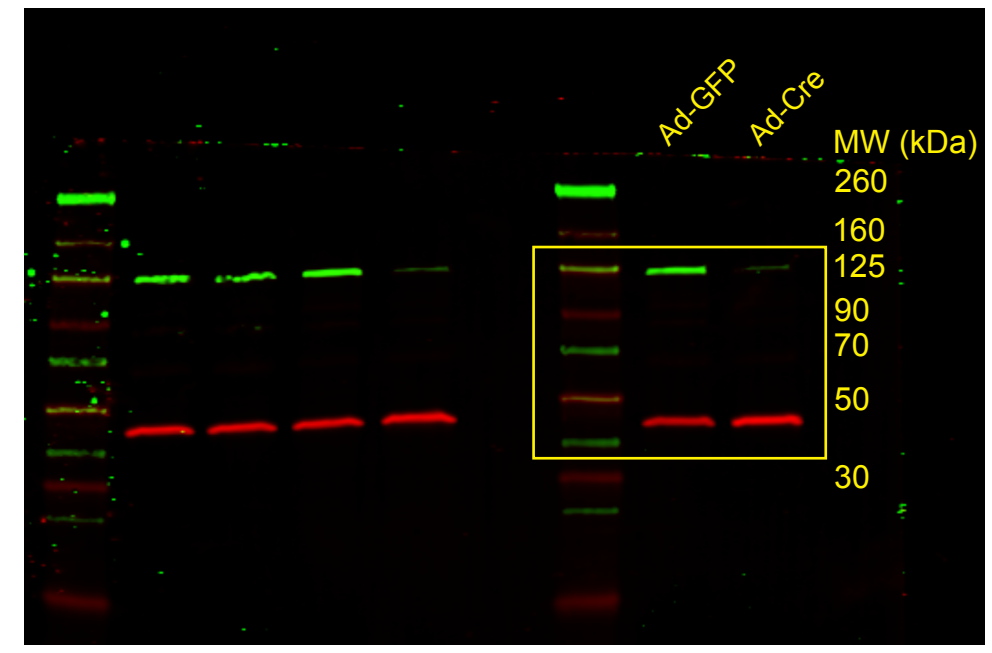

IB: E-cadherin (~120 kDa), β-Actin (42 kDa)

WB for figure S1B

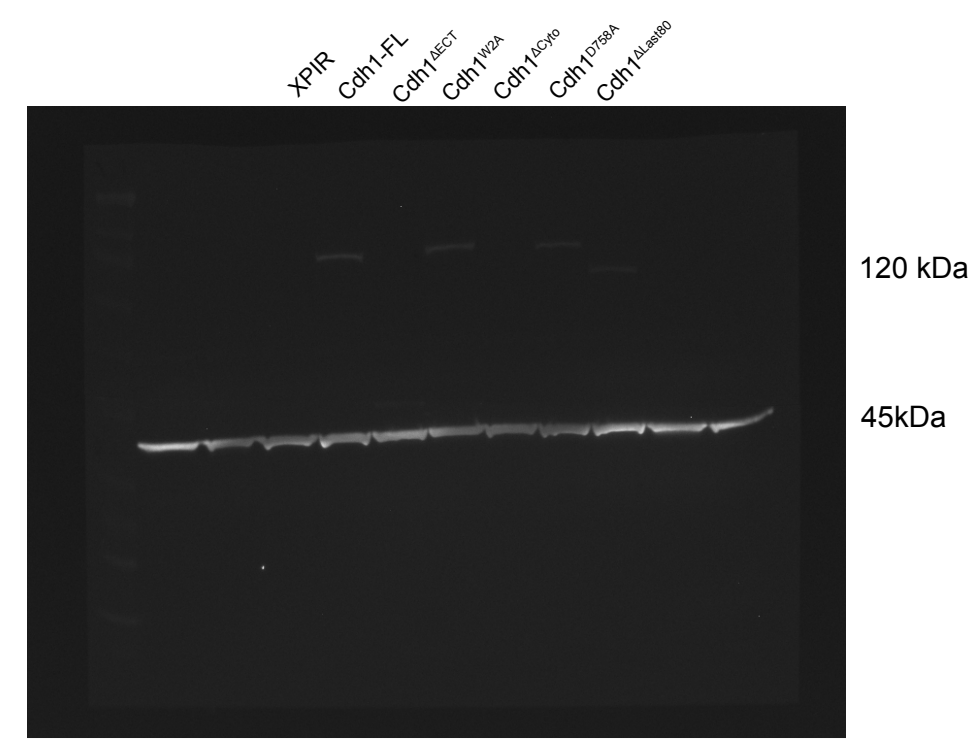

IB: E-cadherin (~120 kDa) + β-Actin (42 kDa)

WB for figure S1G:

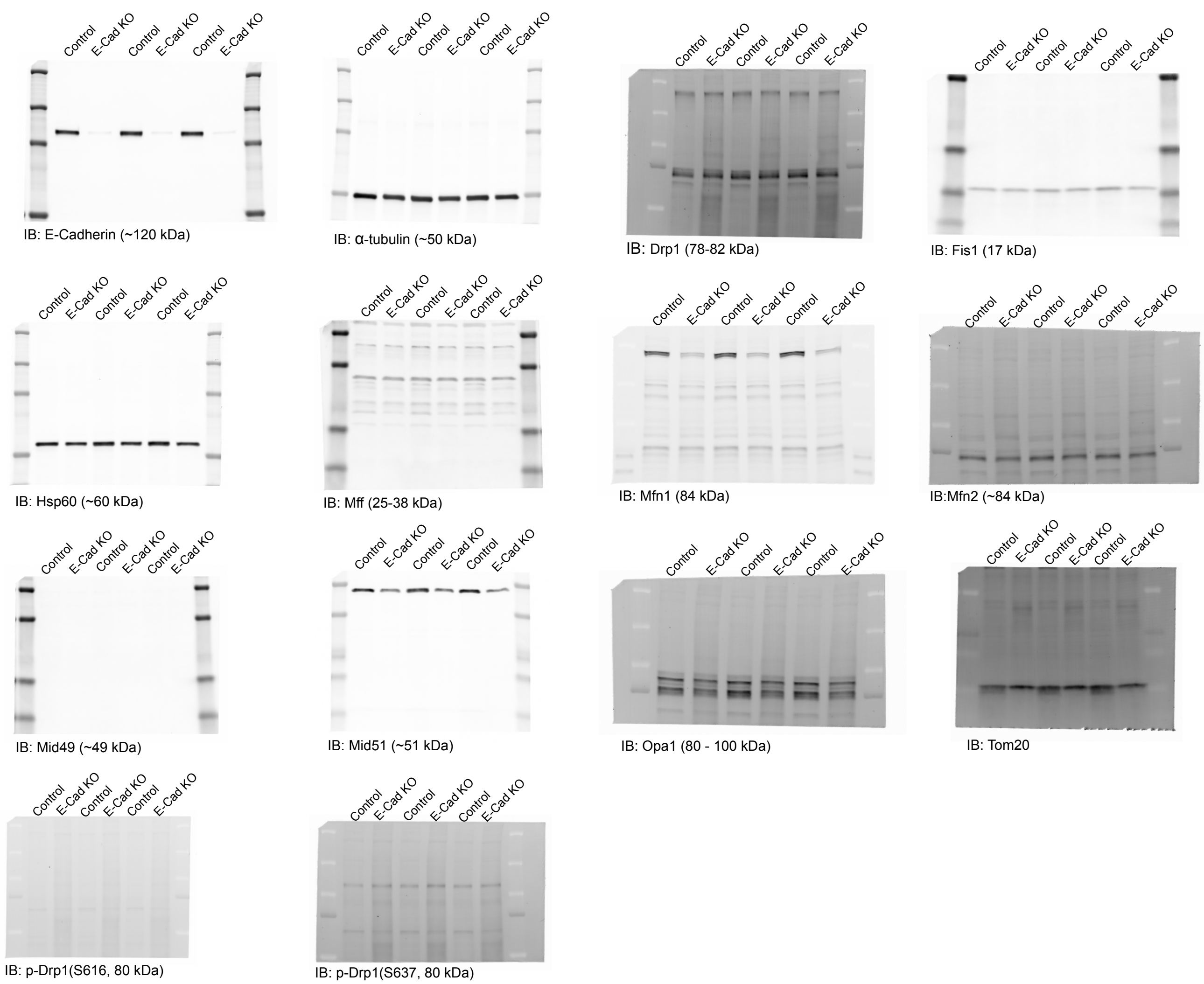

Supplement: Supplementary file 2 [file jcb_202507222_sourcedatafs1.pdf]
